# Effect of Two Different Sugarcane Cultivars on Rhizosphere Bacterial Communities of Sugarcane and Soybean Upon Intercropping

**DOI:** 10.3389/fmicb.2020.596472

**Published:** 2021-01-14

**Authors:** Yue Liu, Huichun Yang, Qi Liu, Xiaowen Zhao, Sasa Xie, Ziting Wang, Ronghui Wen, Muqing Zhang, Baoshan Chen

**Affiliations:** ^1^Guangxi Key Laboratory of Sugarcane Biology, Nanning, China; ^2^College of Agronomy, Guangxi University, Nanning, China; ^3^State Key Laboratory for Conservation and Utilization of Subtropical Agro-bioresources, Guangxi University, Nanning, China; ^4^College of Life Science and Technology, Guangxi University, Nanning, China

**Keywords:** intercropping, sugarcane, microbial community, functional gene, rhizosphere

## Abstract

Intercropping of soybean and sugarcane is an important strategy to promote sustainable development of the sugarcane industry. In fact, our understanding of the interaction between the rhizosphere and bacterial communities in the intercropping system is still evolving; particularly, the influence of different sugarcane varieties on rhizosphere bacterial communities in the intercropping process with soybean, still needs further research. Here, we evaluated the response of sugarcane varieties ZZ1 and ZZ9 to the root bacterial community during intercropping with soybean. We found that when ZZ9 was intercropped with soybean, the bacterial diversity increased significantly as compared to that when ZZ1 was used. ZZ9 played a major role in changing the bacterial environment of the root system by affecting the diversity of rhizosphere bacteria, forming a rhizosphere environment more conducive to the growth of sugarcane. In addition, our study found that ZZ1 and ZZ9 had differed significantly in their utilization of nutrients. For example, nutrients were affected by different functional genes in processes such as denitrification, P-uptake and transport, inorganic P-solubilization, and organic P-mineralization. These results are significant in terms of providing guidance to the sugarcane industry, particularly for the intercropping of sugarcane and soybean in Guangxi, China.

## Introduction

Plant roots are optimal spaces for microbial community aggregation (Turner et al., 2013). Phenotypic traits, such as the structure of roots, may influence microbial recruitment and colonization, depending on microbial cell wall structure, metabolic profile, surface area, nutritional characteristics, and biotic and abiotic stress conditions (Saleem et al., 2018). Plant roots can secrete primary metabolites such as organic acids or amino acids (Vives-Peris et al., 2020); these exudates play an important role in shaping the rhizosphere environment by changing the chemical composition of the soil near the plant roots and by acting as substrate for the growth of selected soil microorganisms (Hu et al., 2018). The composition of plant root exudates changes qualitatively and quantitatively, depending on the plant's nutritional status, growth stage, and even the position of the root in time and space (Backer et al., 2018). These changes in exudates create a strong selection pressure in the rhizosphere, causing plants to drive the selection of specific rhizosphere microbial communities (Huang et al., 2014). Plants naturally select these beneficial microorganisms to aid their own growth and survival, especially under restricted conditions (Lareen et al., 2016). Therefore, differences in root exudates form rhizosphere communities with different functions (Olanrewaju et al., 2019). Modern sugarcane cultivars are derived from highly polyploid hybrids generated by the hybridization of two highly polyploid species (*Saccharum oofficinarum* and *S. spontaneum*) (Meng et al., 2018). Owing to the random sequence of chromosomes in the genome, the chromosome combinations of these hybrids were unique (Grivet and Arruda, 2002). Zhao et al. (2020) demonstrated that sugarcane with different genotypes formed different rhizosphere bacterial communities, and speculated that differences in root exudates were responsible for this difference. The potential for adaptive plant–microbial feedback is particularly relevant for the acquisition of N, an essential nutrient whose availability in agro-ecosystems is controlled by the interaction between fertility management practices and microbial metabolic processes (Schmidt et al., 2019). These interactions affect the structure of the rhizosphere microbial community and create specific network interactions in millimeter-sized habitats that activate rhizosphere nutrients such as P (Chen et al., 2018). The change in microbial structure in soil also affects the process of N and P cycling and further affects the utilization efficiency of N, P, and other nutrients in plants.

Microbial communities play a central role in every biogeochemical cycle on earth, driving the global nutrient cycle through direct feedback on ecosystem function and productivity (Delgado-Baquerizo et al., 2017). Plant growth-promoting rhizobacteria (PGPR) contain a variety of mechanisms that promote growth, including phosphate solubilization, iron carrier formation, biological nitrogen fixation, and the production and activity of 1-amino-cyclopropane-1-carboxylic acid deaminase (Xun et al., 2015; Goswami et al., 2016). These PGPR can affect plant growth and development in different ways. In general, PGPR promote plant growth directly by increasing nutrient acquisition (N, P, K, and essential minerals) or by regulating plant hormone levels, or indirectly, by reducing the inhibitory effects of various pathogens on plant growth and development in the form of biological control agents (Tariq et al., 2017). PGPR also clean the environment by detoxifying pollutants such as heavy metals and pesticides. Studies are continually increasing our understanding of the diversity and importance of soil PGPR communities and their role in improving agricultural sustainability (Santoyo et al., 2016). The impact of PGPR depends on ecological and soil factors, plant species, plant age, stage of development, and soil type. In general, these PGPR regulate plant–soil chemical reactions, paving the way for plant growth and sustainable agriculture (Glick, 2012). Many microorganisms are associated with plants, and the function of the plant microbiome is greater than the sum of the fuctions of its constituent parts, because microbial species often interact strongly with each other and form a complex network (Miller et al., 2018; Rodriguez et al., 2019). Microbial networks typically consist of thousands of interdependent components that interact in reciprocal, synergistic, symbiotic, or parasitic patterns. These interactions have the potential to affect soil fertility and plant health (Van Der Heijden and Hartmann, 2016).

Appropriate intercropping can increase crop yield, and its great achievements in agricultural production are well-known (Nyoki and Ndakidemi, 2016). In particular, the intercropping of soybean and grasses (sugarcane) is also recognized and widely used globally (Solanki et al., 2019). In order to reduce nitrogen leaching and stabilize yield, sugarcane and soybean intercropping has been widely used (Chen et al., 2019a). Even in intercropping, legumes can activate nitrogen fixation (Mahmud et al., 2020). This fixation further improves soil fertility and field ecological conditions, which is conducive to the mutualism of sugarcane and soybean (Romanyà and Casals, 2019). Our previous production data showed that the yield of the sugarcane cultivar, ZZ9, in an intercropping system with soybean was higher than that of ZZ1, and our research revealed that different sugarcane varieties had different rhizosphere bacterial community structures (Zhao et al., 2020). Another previous study confirmed that a sugarcane and soybean intercropping system could change the soil rhizosphere bacterial community structure (Solanki et al., 2017). Therefore, we speculated that different rhizosphere bacterial community structures would be formed when different sugarcane varieties were intercropped with soybean. Many other studies have also demonstrated that different plant cultivars can influence the community structure of bacteria. For example, Huang et al. (2017) showed that there were significant differences in the rhizosphere bacterial structure between the two species of water convolvulus. Jiang et al. analyzed the rhizosphere microbial communities of 12 rabbit eye blueberry varieties and found that plant varieties could influence the rhizosphere bacterial network (Jiang et al., 2017a). Aira et al. (2010) also demonstrated that the community structure of maize rhizosphere microbes was related to plant genotypes. Therefore, it is necessary to study the mechanisms underlying the changes made by different sugarcane varieties to the structure of soil rhizosphere bacterial communities in intercropping systems.

Guangxi is an important sugarcane-growing region in China, accounting for more than 60% of China's total planting area and 68.5% of China's total sugar production (Rukai and Yuan, 2010). Owing to the uneven distribution of perennial rainfall and incomplete irrigation facilities, drought has become one of the main factors restricting the increase in sugarcane yield in Guangxi, and the breeding and promoting of drought-resistant varieties are urgent for improving sugarcane production (Santos et al., 2019). “Zhongzhe” are new sugarcane cultivars selected and bred by the State Key Laboratory for Conservation and Utilization of Subtropical Agro-bioresources, and ZZ1 and ZZ9 are two varieties of “Zhongzhe” (Yin et al., 2020). Both ZZ1 and ZZ9 exhibit high resistance to smut with strong persistent roots and have been planted in many areas of Guangxi (Zhang et al., 2019). These varieties have great advantages in yield and can replace the sugarcane varieties currently planted by farmers. Previous studies have found that ZZ1 and ZZ9 have differences in water-use efficiency, which also indicates differences in nitrogen use (Araus et al., 2020). Therefore, ZZ1 and ZZ9 may have different patterns when intercropped with soybean, and it is worth conducting an in-depth exploration of the intercropping benefits of these two varieties. In this experiment, we compared sugarcane and soybean intercropping with different sugarcane cultivars (ZZ1 and ZZ9) to determine suitable intercropping varieties. Therefore, the central questions of this study were: (1) Does the intercropping of ZZ1 and ZZ9 with soybean have different effects on the rhizosphere microbial community? (2) In the intercropping system, do ZZ1 and ZZ9 play a dominant role in forming a similar rhizosphere bacteria structure to monocropping? (3) What is the relationship between the rhizosphere bacterial community structure and crop growth? Our study aimed to answer the above questions, and provide new ideas for the efficient use of sugarcane fields and the sustainable development of the sugarcane industry.

## Materials and Methods

### Plants and Field Experiment Design

The study was conducted in Quli, Fusui (107°31′ to 108°06′ E and 22°17′ to 22°57′ N) forage breeding farm of Guangxi University in the summer of 2018. The experiment was based on the sugarcane intercropping demonstration project in Guangxi, covering an area of 0.3 km^2^. The average annual temperature was 21.3°C. The lowest temperature of 2017 was −0.6°C and the highest was 39.5°C. The annual total radiation was 108.4 kcal/cm, the annual average sunshine duration was 1,693 h, and the frost-free period was 346 days. The annual precipitation of the whole region was between 1,050 and 1,300 mm. The soil in the long-term sugarcane field had the following characteristics: laterite, pH value 5.15, organic matter 19.47 g/kg, total nitrogen 0.84 g/kg, total phosphorus 2.98 g/kg, total potassium 7.11 g/kg, 136 mg/kg alkaline hydrolyzed nitrogen, 83 mg/kg available phosphorus, and 77.1 mg/kg available potassium. In this study, two modes of interactivity, ZZ1–soybean and ZZ9–soybean, were set up. ZZ1 and ZZ9 are two new “Zhongzhe” series lines selected and bred in the state key laboratory for conservation & utilization of subtropical agro-bioresources. Their excellent characteristics can be seen from the field agronomic data of the two varieties (Supplementary Figure 1): ZZ9 was significantly better than ZZ1 in terms of plant height and stem thickness; and also had a stronger root system. Both of them were from the same parent (ROC 25 × Yunzhe 89-7) (Yin et al., 2020). In the case of soybean, we used the local “GUIZAO2” cultivar. “GUIZAO2” is a high-yield variety bred locally in Guangxi with the characteristics of drought resistance, adaptation to local climate, and shade tolerance. This cultivar is suitable for intercropping with sugarcane and is widely planted in Guangxi (Huaizhu et al., 2004). Each intercropping pattern was set with three replicates. The experiment consisted of a total of six blocks. There were three blocks with ZZ1–soybean and three with ZZ9–soybean, each covering 30 m × 42 m. Each block consisted of 12 rows of sugarcane and 12 rows of soybeans planted alternately, each row spaced 1.2 m apart (Supplementary Figure 2A).

### Soil Sample Collection and Physicochemical Analysis

The roots of each plant were separated from the soil, the loosely attached soil was removed by manual shaking, and the soil adhering to the roots was collected as rhizosphere soil (Wang et al., 2018). The soil collected between the two rows of sugarcane was defined as mono_sug (Supplementary Figure 2B). Accordingly, the rhizosphere soil collected from ZZ1 roots was defined as mono_sug1, and the rhizosphere soil collected from ZZ9 roots was defined as mono_sug9. The rhizosphere soil from sugarcane collected from the root surface near the soybean side was defined as inter_sug, and the rhizosphere soil of soybean that was collected from the root surface near the sugarcane side was defined as inter_soy. Accordingly, rhizosphere soil collected from the ZZ1–soybean intercropping pattern was defined as inter_soy1 and inter_sug1, and rhizosphere soil collected from the ZZ9–soybean intercropping pattern was defined as inter_soy9 and inter_sug9. Each sample consisted of three sampling blocks, and from each block, six sugarcane and six soybean rhizosphere soil samples were collected (Supplementary Figure 2C). The roots of plants were vigorously shaken to remove soil that was not tightly bound to the roots. The soil from each block was then mixed, and plant debris and stones were removed with a 2 mm sieve. Each soil sample was divided into three parts for DNA extraction, environmental factor determination, and soil enzyme activity determination. Soil organic carbon (SOC), total nitrogen (TN), and available phosphorus (AP) content was then measured as previously described (Bremner and Tabatabai, 1972; Soon and Abboud, 1991; Zhaolei et al., 2017), and soil enzyme kits were used to determine soil urease (S-U), soil sucrase (S-SC), soil catalase (S-CAT), and soil acid phosphatase (S-ACP) (Wang et al., 2014; Hou et al., 2020). Each soil sample was analyzed in three replicates, 0.5 g per sample.

### DNA Extraction, Amplicon Generation, and High-Throughput Sequencing

DNA was extracted using the E.Z.N.A soil DNA kit (Omega Bio-Tek, Inc., Norcross, GA, USA) for the corresponding samples. The concentration and purity of the extracted DNA were measured using NanoDrop One spectrophotometer (Thermo Fisher Scientific, MA, USA). PCR reaction mixtures—containing 25 μL of 2 × Premix Taq (Takara Biotechnology, Dalian Co., Ltd, Dalian, China), 1 μL of each primer (10 mM), and 3 μL of template DNA (20 ng/μL) in a total volume of 50 μL—were amplified by thermocycling under the following conditions: 5 min at 94°C for initial denaturation, followed by 30 cycles of 30 s each for denaturation at 94°C, 30 s for annealing at 52°C, 30 s for extension at 72°C, and then a final extension at 72°C for 10 min. The V3–V4 regions of the bacterial 16S rRNA gene were amplified using the primers 338 F (5′-ACTCCTACGGGAGGCAGCA-3′) and 806R (5′-GGACTACHVGGGTWTCTAAT-3′) (Hong et al., 2015). Amplification was performed using a BioRad S1000 thermocycler (Bio-Rad Laboratories, CA, USA). Subsequently, DNA libraries were constructed with the Illumina TruSeq DNA Sample Preparation Kit (Illumina, San Diego, CA, USA). High-throughput sequencing of 16S rRNA genes was carried out using an Illumina HiSeq2500 platform and 250 bp paired-end reads were generated (Guangdong Magigene Biotechnology Co., Ltd., Guangzhou, China).

### Statistical and Bioinformatics Analysis

Using the Mothur software (V1.35.1), raw tag sequences were assigned to unique barcodes and primers to obtain clean read tags, which were then assembled using the software FLASH (V 1.2.7, http://ccb.jhu.edu/software/FLASH/) (Magoč and Salzberg, 2011). Sequence analysis was performed using the USEARCH software (V8.0.1517, http://www.drive5.com/usearch/) (Edgar, 2010). To determine operational taxonomic units(OTUs), the 16S rRNA gene sequences were trimmed to a fixed length of 360 bp, sorted by abundance, de-replicated and then clustered together using UPARSE (version 7.1 http://drive5.com/uparse/) (Edgar, 2013). Using UCHIME (v4.2.40 http://www.drive5.com/uchime) (Edgar et al., 2011), chimeric sequences were screened against the GOLD database (release 115; https://www.arb-silva.de/) and removed (Danielsson et al., 2019). Sequences with ≥97% similarity were assigned to the same OTU. Taxon assignment was performed using the SILVA v123 database (Quast et al., 2012).

Alpha diversity was analyzed using QIIME (V1.9.1) to assess the complexity of species diversity in the samples and was displayed using R (V2.15.3). The diversity of the samples was estimated by determining the Chao 1 and Shannon indexes. At the OTU level, we examined the differences in three sample types (ZZ1, ZZ9, and GUIZAO2 monocropping) and two intercropping patterns (ZZ1–soybean and ZZ9–soybean) in observed species richness. Beta diversity analysis was used to evaluate differences in the species complexity of samples. For the general analysis, the BioConductor package edgeR (version 3.8.5) was used to normalize the filtered OTU sequence count for each bacterial taxon by the “trimmed means of M” (TMM), and the normalized count was expressed as the relative abundance “count per million” (CPM) (Hartman et al., 2018). We used the R software and Local Perl scripts to generate sample distance heat maps based on the UniFrac distance matrix. Principal co-ordinate analysis (PCoA) was then used to display differences in the species composition of microbial communities. We examined the effects of sample type and intercropping patterns on community dissimilarity using permutational analysis of variance (PERMANOVA). Furthermore, Pearson correlation analysis was used to find the relationship between alpha diversity and environmental factors, and we used the Mantel test to study the relationship between beta diversity and environmental factors. The Mantel test, PCoA, and distance-based redundancy analysis (dbRDA) were performed using the “vegan” package in R v3.6.3 (Bargaz et al., 2017).

### Cropping-Sensitive Rhizobacterial OTUs (CsOTUs) in Response to the ZZ1 and ZZ9 Intercropping Patterns

For an in-depth analysis of the effects of different intercropping patterns on root and soil microbial communities, we used a complementary approach to determine the OTUs responsible for the observed effects (Hartman et al., 2018), i.e., a correlation-based index species analysis and the R package index to calculate the point-binary correlation coefficient (R) of positive correlations between OTUs and one or more planting systems. Differences in OTU abundance within soil and root communities were obtained using the likelihood ratio tests (LRT) in the R package “edgeR” with the same table of OTU thresholds, comparing the cropping systems with respect to both kingdoms (Robinson et al., 2010). The abundance of an OTU was determined as the false discovery rate (FDR) correction between one or more planting systems with *p* < 0.05, considered to be indicative of a response to ZZ1 and ZZ9 intercropping patterns. Thereafter, we defined the OTUs confirmed by indicator type analysis and LRT as crop-sensitive OTUs (CsOTUs).

### Co-occurrence Network Analysis

For an in-depth assessment of soil and root bacterial communities, we performed analyses to find the Spearman's rank correlation coefficient for all pairs of bacteria to determine topological network properties. These included the total number of network nodes (OTUs), the total number of edges (connections between nodes representing significant positive correlations between OTUs), and the degree of co-occurrence (number of direct correlations to a node). For this, we incorporated TMM-normalized CPM bacterial counts into separate OTU tables for soil and root communities. Spearman rank correlation analyses were performed to assess associations between all bacterial OTU pairs. We calculated the aforementioned network properties and, to examine the community structure in the soil and root element networks, we identified the network modules from the substructures of the nodes, where the edge density within the groups is higher than that between them (Hartman et al., 2018). Microbial taxa that repeatedly co-occur with other taxa in microbial co-occurrence networks are considered to be ecologically relevant and to play important roles in the microbiome (Agler et al., 2016; Van Der Heijden and Hartmann, 2016; Hartman et al., 2018). We identified the key soil and root metanetwork OTUs, defining these as each network node degree value within the top 1% of the node. We prioritized this simple definition over a more complex approach (e.g., based on high degree and low betweenness centrality) because both definitions uncovered largely the same sets of keystone OTUs, which were identified separately for sugarcane and soybean. The Mantel Test was used to determine the relationship between modules and environmental factors.

### Prediction of the Function of the Rhizosphere Microbiome

PICRUSt2 (Phylogenetic Investigation of Communities by Reconstruction of Unobserved States 2), a software based on the marker gene sequence to predict functional abundance, was used to predict the function of the rhizosphere microbiome (Douglas et al., 2020a). PICRUSt2 contains a newer, larger database of gene families and reference genomes, interoperates with any operational classification unit (OTU) screening or denoising algorithms, and is capable of phenotypic prediction. Benchmarks show that PICRUSt2 is generally more accurate than PICRUSt and other competing methods (Douglas et al., 2020b). We used it to mark the function of rhizosphere bacterial communities under the ZZ1 and ZZ9 intercropping patterns. In each dataset, all predicted gene families and N and P cycle pathways were compared to those from metagenome sequencing in terms of their Kyoto Encyclopedia of Genes and Genomes (KEGG) annotations that were downloaded from the KEGG website (Baquiran et al., 2020). R was used to analyze the correlation between functional genes and rhizosphere, and the contribution of corresponding bacterial populations to functional genes was summarized.

## Results

### Rhizosphere Diversity of ZZ1 and ZZ9 Under Intercropping With Soybean

**Figure 1:**
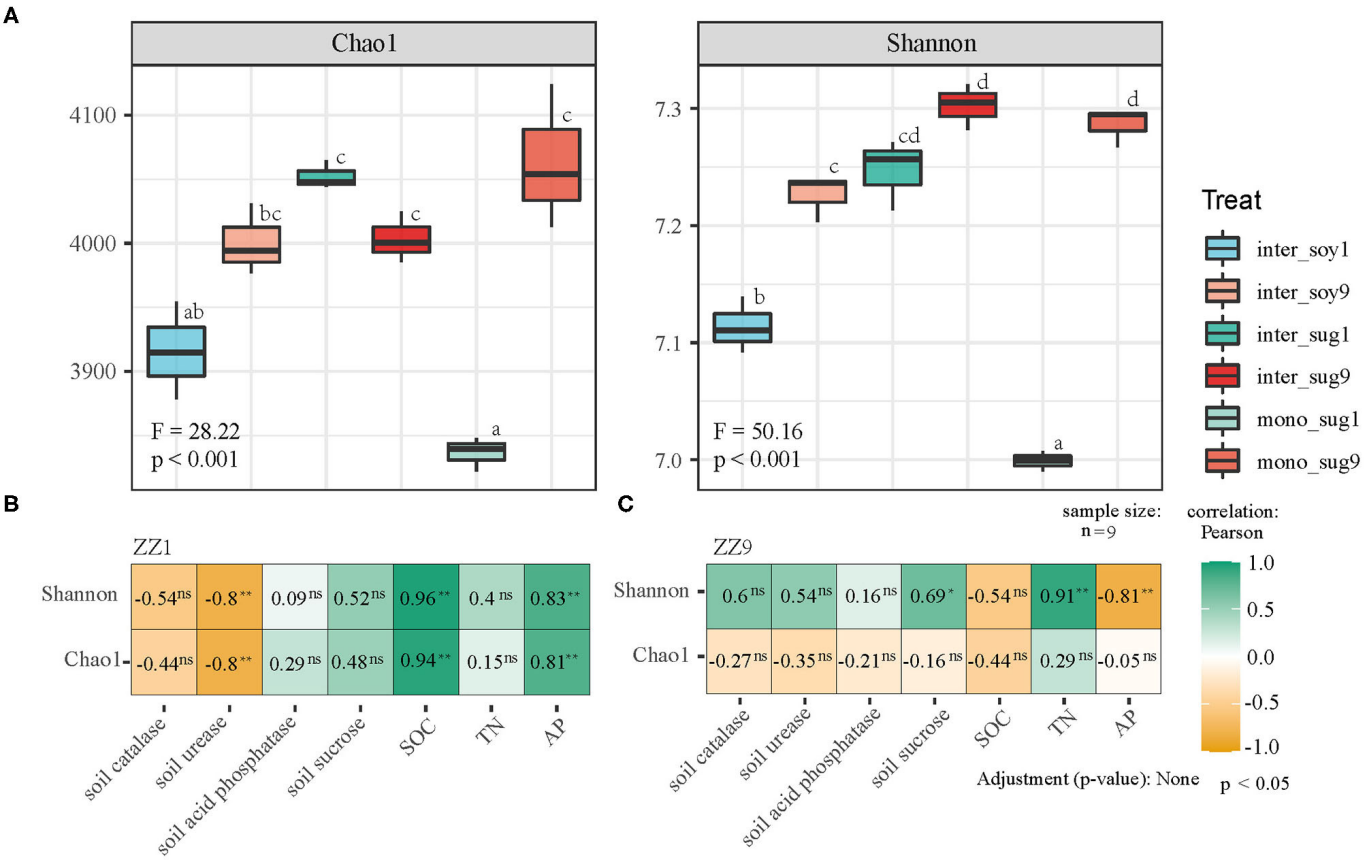
**(A)** Bacterial alpha-diversity measurements of represented by Chao 1 and Shannon indexes in each pattern. F, Fisher's F-ratio; *p, p*-value. Different letters next to the bars represent significant differences between the measured indices. **(B,C)** The correlation between bacterial alpha-diversity and environmental factors using Pearson analysis. *P* > 0.05 (NS), *P < 0.05, **P < 0.01.

Alpha diversity analysis indicated that mono_sug1 and mono_sug9 differed significantly, and mono_sug9 had higher Chao1 and Shannon indexes (<0.001). As compared to intercropping with ZZ1, soybean intercropping with ZZ9 had higher Chao1 and Shannon indexes (<0.001), but the difference between inter_sug1 and inter_sug9 was not significant (Figure 1A). In soil characteristics, there were significant differences in SOC, TN, and AP. When considering the whole planting system (including inter_soy, mono_sug, and inter_sug), there were significant differences in S-U and S-SC between ZZ1 and ZZ9 intercropping patterns (Table 1). Pearson correlation analysis showed that ZZ1 Chao1 and Shannon indexes of the rhizosphere and S-U content were significantly negatively correlated, while SOC and AP content were significantly positively correlated with the indexes (Figure 1B, ρ < 0.05); on the other hand, ZZ9 Shannon index and TN content were significantly positively related, whereas AP levels were significantly negatively correlated with the index (Figure 1C). ZZ1 and ZZ9 and soil catalase (S-CAT) and acid phosphatase (ACP) had no significant relationship.

**Table 1:** Soil characteristics of different patterns.

**Sample**	**S-CAT (U/g)**	**S-U (U/g)**	**S-ACP (μmol/d/g)**	**S-SC (U/g)**	**SOC (g·kg^**−1**^)**	**TN (g·kg^**−1**^)**	**AP (g·kg^**−1**^)**
Inter_soy1	10.96 ± 0.49a	294.34 ± 28.49ab	56.15 ± 4.26a	54.38 ± 0.03a	14.14 ± 0.71ab	1.22 ± 0.06bc	1.80 ± 0.26bc
Mono_sug1	12.45 ± 0.37a	363.53 ± 37.79b	53.82 ± 1.76a	54.58 ± 0.16a	11.19 ± 0.01a	1.12 ± 0.06b	1.02 ± 0.03a
Inter_sug1	11.31 ± 054a	252.87 ± 23.51a	56.07 ± 4.46a	54.84 ± 0.20a	18.22 ± 0.89cd	1.18 ± 0.05b	1.89 ± 0.04bc
Inter_soy9	11.47 ± 1.80a	382.01 ± 30.84b	57.00 ± 3.39a	54.55 ± 0.67a	21.22 ± 1.63d	0.92 ± 0.02a	2.00 ± 0.08c
Mono_sug9	11.54 ± 0.92a	363.90 ± 22.11b	57.61 ± 3.32a	62.47 ± 4.11b	14.48 ± 0.79ab	1.37 ± 0.09c	1.82 ± 0.02bc
Inter_sug9	22.49 ± 1.70b	487.65 ± 34.50c	62.41 ± 3.17a	93.01 ± 1.31c	17.59 ± 1.43bc	1.55 ± 0.00d	1.56 ± 0.01b
F	31.87	14.41	1.337	148.3	23.03	33.87	20.25
*P*	<0.001	<0.001	=0.313	<0.001	<0.001	<0.001	<0.001

*F, Fisher's F-ratio*.

*p, p-value*.

**Figure 2:**
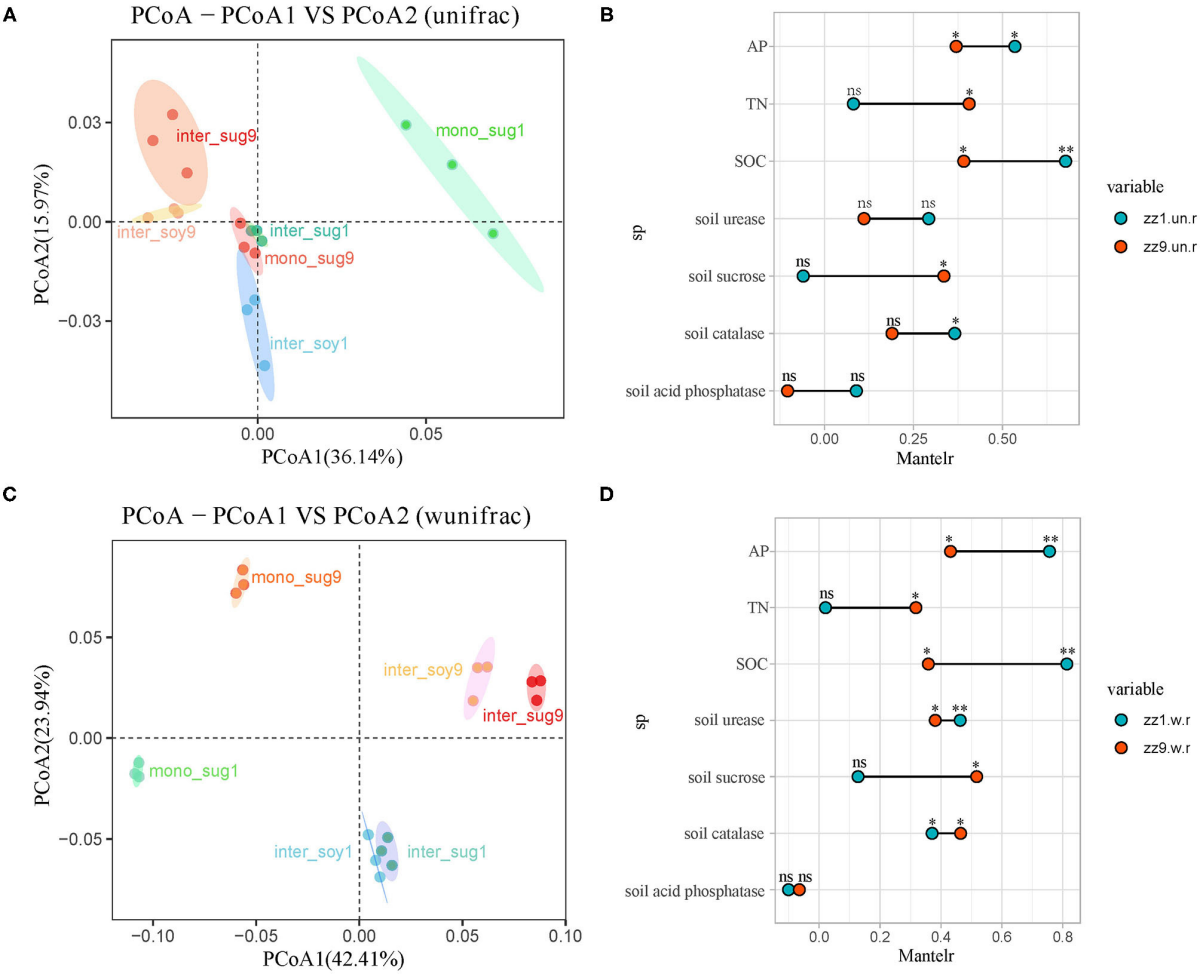
**(A,C)** Principal coordinate analyses (PCoAs) using UniFrac distance matrix, to analyze the differences in the diversity of bacterial beta under different patterns, **(A)** is unweighted, **(C)** is weighted. **(B,D)** Mantel test was used to o compare the correlation difference of soil nutrients between ZZ1 and ZZ9, **(B)** is unweighted, and **(D)** is weighted. *P* > 0.05 (NS), *P < 0.05, **P < 0.01.

In terms of beta diversity, phylogenetic analysis of bacterial community members and composition using unweighted and weighted UniFrac distance showed that ZZ9 had more similar community composition than ZZ1, and the correlations among inter_sug9, mono_sug9, and inter_soy9 were closer than that of ZZ1, while mono_sug1 in ZZ1 was significantly different from inter_sug1 and inter_soy1 (Figure 2A). After weighting, there was a significant difference in the soil composition between ZZ9 and ZZ1 when abundance was considered, and compared with unweighted results, mono_sug9, inter_soy9, and inter_sug9 were significantly different (Figure 2C). The results of the Mantel test showed that the diversity index of ZZ1 was significantly correlated with AP, SOC, S-U, and S-CAT, whereas that of ZZ9 was significantly correlated with TN and S-SC (Figures 2B,D).

**Figure 3:**
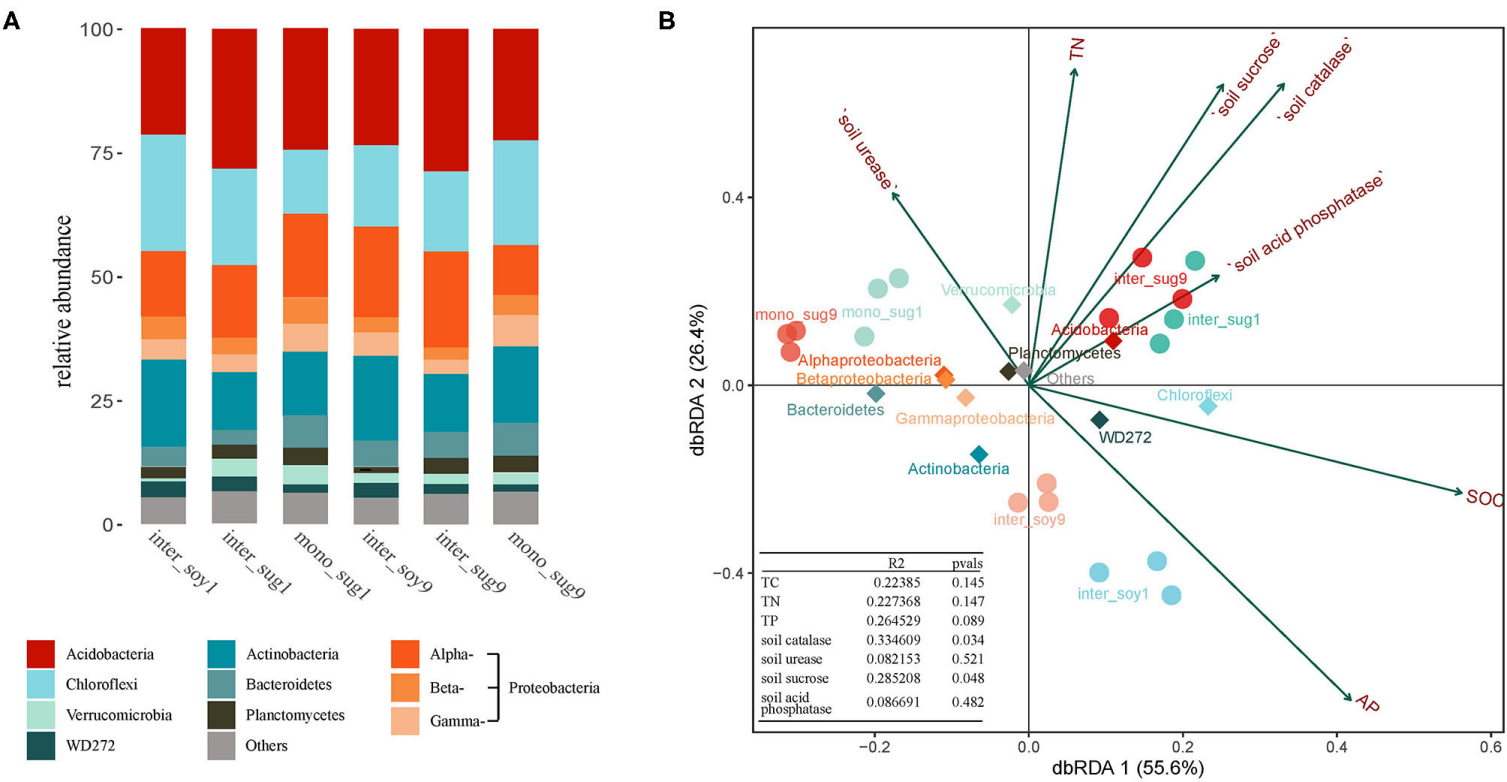
**(A)** Relative abundance of different phyla in each pattern. **(B)** Distance-based Redundancy analysis of different patterns (dot), abundant classes (rhombus), and environmental factors (arrows) indicates the dominant communities and influential environmental factors.

The dominant bacteria (relative abundance >1%) with relatively high contributions are listed in Supplementary Table 1. Proteobacteria had the highest relative abundance of 25.24% (Alphaproteobacteria 16.42%, Betaproteobacteria 5.02%, and Gammaproteobacteria 3.80%). Following this were Acidobacteria (24.89%), Chloroflexi (17.29%), Actinobacteria (14.49%), Bacteroidetes (4.62%), Verrucomicrobia (2.63%), Planctomycetes (2.48%), and WD272 (2.35%). Sugarcane varieties used for intercropping had a significant influence on dominant bacterial species. Among these, as compared to inter_sug1, the relative abundance of Alphaproteobacteria in inter_sug9 was higher, while Chloroflexi had a higher relative abundance in the ZZ1–soybean intercropping pattern (Figure 3A). The correlation between environmental factors and bacterial composition was further explored by dbRDA analysis, and the first two axes of dbRDA accounted for 55.6 and 26.4%, respectively, of the total variation of the data (Figure 3B). AP correlated with the bacterial community distribution of inter_soy9 and inter_soy1 soils. S-ACP and SOC were related to the bacterial community distribution of mono_sug9 and mono_sug1 soils. In addition, S-ACP correlated with inter_sug1 and inter_sug9, while SOC correlated with the phyla Chloroflexi and WD272.

### Co-occurrence Network Analysis of ZZ1 and ZZ9 in Intercropping Patterns

**Figure 4:**
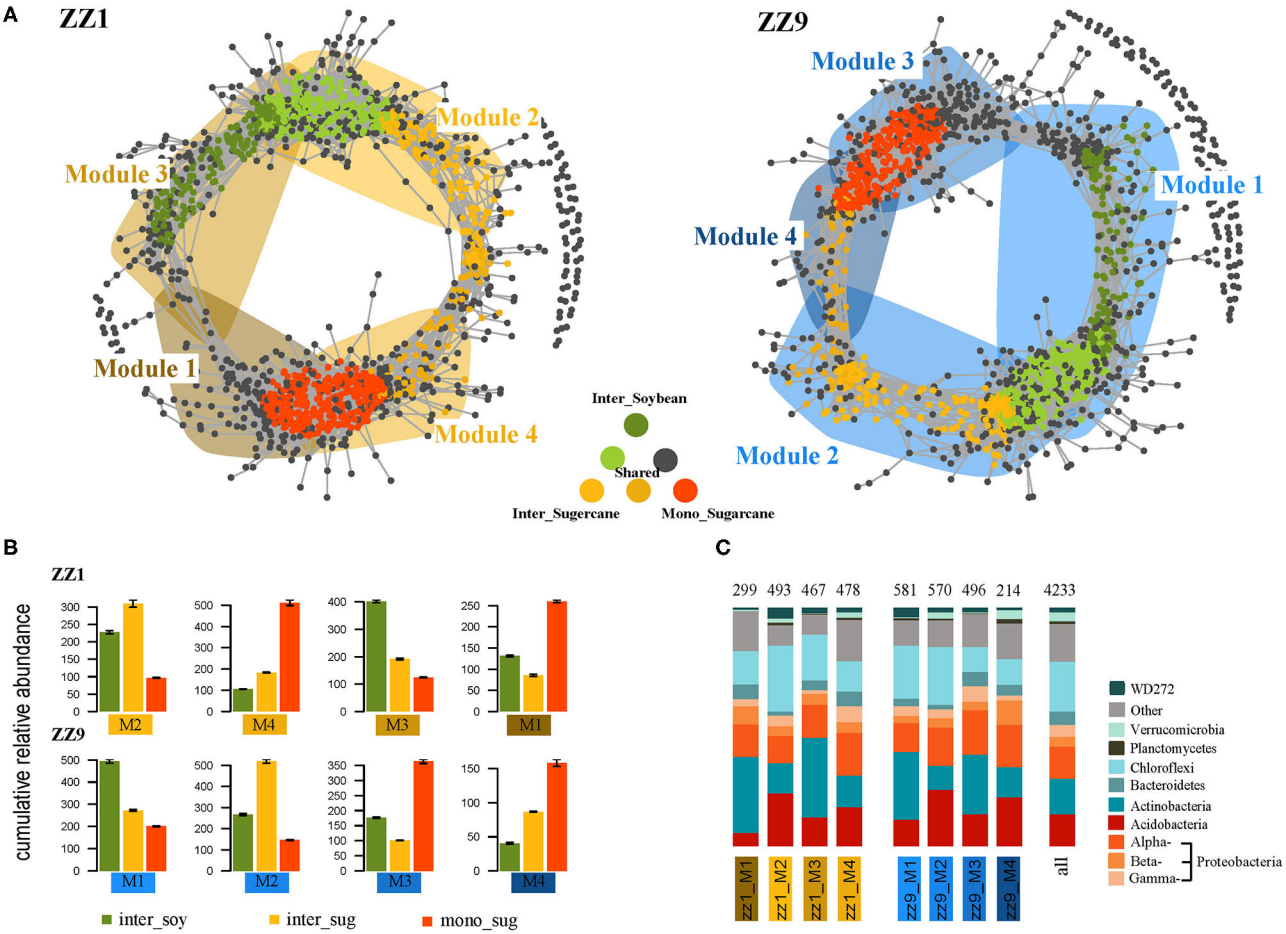
**(A)** Co-occurrence networks visualizing significant correlations (ρ > 0.7, *p* < 0.001; indicated with gray lines) between bacteria OTUs in ZZ1 and ZZ9 communities. Circles indicate bacteria, OTUs are colored by their association to the different cropping systems (gray OTUs are insensitive to variety difference). Shaded areas represent the network modules containing csOTUs. **(B)** Cumulative relative abundance (as counts per million, CPM; y-axis in ×1,000) of all bacteria of the cropping sensitive modules in soil and root networks. The cumulative relative abundance in samples of Inter_Soybean (dark green), Inter_Sugercane (orange), Mono_Sugarcane (red) cropping systems indicates the overall response of cropping sensitive modules to the different patterns. **(C)** Qualitative taxonomic composition of cropping sensitive modules is reported as proportional OTUs numbers per bacterial class and compared to the overall taxonomic distribution in the entire dataset (column “all”).

Next, we explored the distribution of bacterial symbiotic patterns of csOTUs in soil and root communities. In Figure 4A, the dots represent bacteria and are colored according to their association with different cropping systems; the gray dots represent insensitivity to experimental treatments. The shaded region represents the network module containing each csOTU. In the intercropping pattern of ZZ1 and soybean, module 1 (M1) and module 4 (M4) contained OTUs corresponding to mono_sug1, while module 2 (M2), and module 3 (M3) mainly represented intercropping treatment and contained almost all the csOTUs in the intercropping pattern. For ZZ9, M3 and M4 contained OTUs corresponding to mono_sug9, while M1 and M2 mainly contained intercropping csOTUs. OTUs that corresponded to inter_sug1 were distributed mostly in ZZ1, and some csOTUs were not even included in the main modules, whereas in ZZ9, inter_sug9, and inter_soy9 were more centrally contained in M1 and M2 (Figures 4A,B). In addition, the modules representing intercropping systems in ZZ9 had higher cumulative relative abundance than those in ZZ1 (Figure 4B), which can also be seen in Figure 4C, where the comparison of OTUs (in terms of number of taxonomic components in each module) is provided. Analysis of key species in rhizosphere soil of ZZ1 and ZZ9 found that all key species in the ZZ1 intercropping pattern appeared in inter_soy1 (Supplementary Table 2), while in the ZZ9 intercropping pattern, key species appeared in inter_sug9 (Supplementary Table 3). It is worth noting that more than half of the key species of ZZ9 belonged to Acidobacteria, while the key species of ZZ1 were rather complex, including Proteobacteria, Actinobacteria, Planctomycetes, Acidobacteria, Chloroflexi, and Bacteroidetes. In the node degree analysis, mono_sug1 and inter_soy1 had a higher node degree in ZZ1 than in ZZ9, where inter_sug9 had a higher node degree (Supplementary Figure 3). In addition, the results of the Mantel test further showed that, in the two sugarcane–soybean intercropping systems, the correlation between ZZ9 and soil environmental factors was higher, and it was related to all other factors except for S-ACP, but ZZ1 was only related to AP, SOC, SU, and S-CAT (Supplementary Figure 4).

### Relationship Between Functional Genes of Soil Bacterial Community and Rhizosphere Soil Nutrients

**Figure 5:**
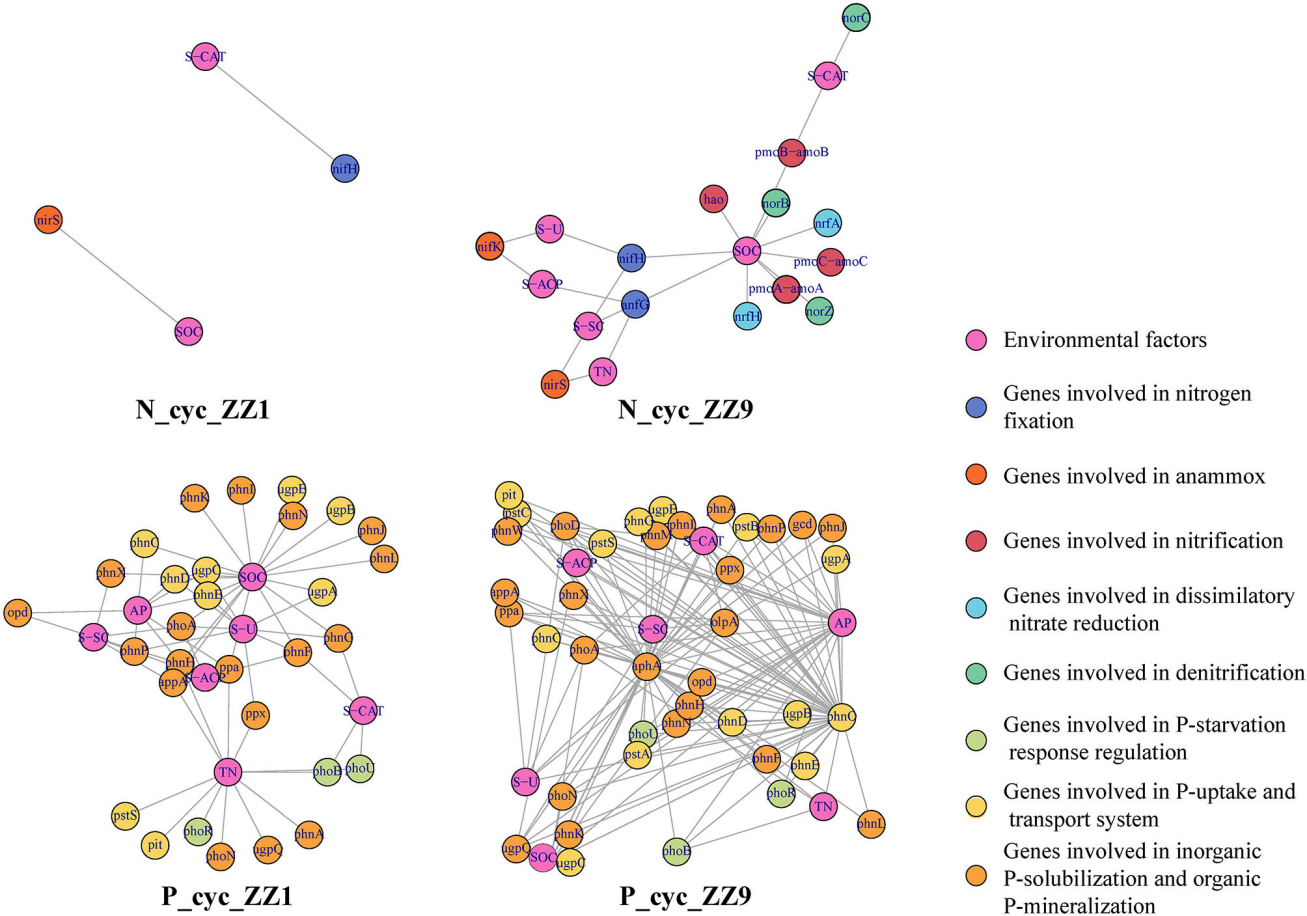
Relationship between functional genes with N, P cycling pathways in soil under two Intercropping Patterns. Pink dots represent environmental factors, and other colors represent the function of functional genes in the N, P cycle.

For the representative sequences of csOTUs in the co-occurrence network analysis, the genes related to the N and P cycle pathways were identified, and the PICRUSt2 was used for functional annotation to determine the microbial community functions of each csOTU; these functional genes were then associated with corresponding environmental factors. In Figure 5, pink circles represent each environmental factor, and other circles of different colors represent different gene functions. We found that both the N and P cycle pathways had more genes in response to environmental factors in the intercropping system of ZZ9 than in ZZ1. In the N cycle pathway, ZZ1 had only two genes, *nifH* and *nirS*, which were, respectively, associated with S-CAT and SOC. However, combined with the representative quality of each gene, we found that the contribution of these two genes was low; in contrast, ZZ9 was associated with more functional genes, such as *norB, norC*, and *norZ* (Supplementary Figure 5), which had a higher contribution, mainly related to denitrification. In the P cycle, ZZ9 had more genes related to the P-uptake and transport system, inorganic P-solubilization, and organic P-mineralization. In addition, the analysis of the bacterial contribution of functional genes related to ZZ9 and ZZ1 in the N and P cycle pathways found quite evident differences in the two sugarcane varieties (Supplementary Figure 6 and Supplementary Figure 7). The relationship between functional genes and each group in the N and P cycling pathways was further studied. PCA results showed that inter_sug1 and inter_sug9 were significantly different in the N cycle pathway and were mainly influenced by several genes such as *nrfA, nrfH, napB*, and *norZ*. There was a significant difference between inter_soy1 and inter_soy9 in the P cycle pathway, mainly as a result of some inorganic P-solubilization and organic P-mineralization genes. The distinction between inter_sug1 and inter_sug9 was also large, mainly as result of differences in P-uptake and transport system related genes. Significant differences were found in the repertoire of putative functional genes in the microbiota between the ZZ1 and ZZ9 varieties. No matter whether it was the N or P pathway, inter_sug9 and mono_sug9 were closely related (Supplementary Figure 7). However, inter_sug1 and mono_sug1, in the intercropping pattern between ZZ1 and soybean, differed from each other.

## Discussion

### Different Bacterial Community Structures Are Formed in the Intercropping System of ZZ1 and ZZ9

Through alpha diversity analysis, we found that the bacterial population diversity and abundance were significantly higher in ZZ9 than in ZZ1 (Figure 1A), which meant that the relationship between bacteria and the soil environment was more complex and the ecosystem was more stable in ZZ9 (Yang et al., 2018a). Weidner et al. (2015) suggested that high soil microbial diversity is favorable for positive plant-soil feedback and N nutrient supply in soil. Differences in biomass and root morphology among different cultivars of the same species may cause differences in the microbial diversity and abundance (Hui et al., 2017; Chen et al., 2019b). For example, Jiang et al. (2017b), suggested that high-yielding cultivars have significantly increased root porosity and an abundance of specific microorganisms, indicating that their large roots and porous soil facilitate gas exchange in the rhizosphere soil, thereby affecting the rhizosphere bacterial community. This can also be seen from the field agronomic data of the two varieties in this study (Supplementary Figure 1): ZZ9 exhibited significantly better plant height and stem thickness, as well as a stronger root system than ZZ1. Bacterial communities may also be affected by the growth of fine roots, which have a much greater surface area than large roots and may carry more nutrients and metabolites. These properties could make them prime sites for proliferation of microbial colonies. This indicates, in part, that plants with bigger roots may be able to associate with a highly diverse microbial community for mutual benefit (Gaiero et al., 2013). This seems to be reflected in the roots of ZZ9 (Supplementary Figure 9).

The results of the beta diversity analysis showed that the soil components were more closely related to ZZ9 than to ZZ1 under the intercropping pattern of ZZ9 and soybean (Figure 2A), indicating that ZZ9 was more closely related to the soybean root system. After adding species abundance weighting, the difference in bacterial community structure between ZZ1 and ZZ9 could be seen more clearly (Figure 2C), and it indicated that intercropping with soybean changed the relative abundance of bacteria in sugarcane rhizosphere, which could be explained by the difference in quantity and composition of their root exudates (Aulakh et al., 2001). Most of these secretions are primary metabolites such as carbohydrates, amino acids and organic acids (Chaparro et al., 2013), which are secreted by the roots of plants and spread to the soil, affecting the content of nutrients such as C and N in the rhizosphere soil, and thus enriching the microbial community (Vives-Peris et al., 2020). Zhao et al. (2020) indicated that the root exudates of sugarcane with different genotypes had specific effects on the formation of different rhizosphere bacterial communities; they, thus speculated that root exudates of sugarcane could affect the recruitment of bacterial communities in rhizosphere thereby enhancing plant resistance. In addition, it also showed that intercropping with soybean could change the relative abundance of the sugarcane rhizosphere bacterial community and form a different rhizosphere bacterial community structure from that of single cropping sugarcane (Paungfoo-Lonhienne et al., 2017). Since the phenotypic structure of the root system had an impact on water absorption, the available N was mostly soluble in water, so many root structural features that promote water absorption (such as root length and diameter) could also generally improve the absorption rate of N (Araus et al., 2020).

Through the analysis of the dominant bacteria in the soil components, it was found that the intercropping varieties had significant influence on the dominant bacteria species. Chloroflexi was relatively more abundant in the treatment of soybean and ZZ1 intercropping as compared to ZZ1 monocropping. In the ZZ9–soybean intercropping pattern, the relative abundance of Alphaproteobacteria was higher than that of ZZ9 monocropping (Figure 3A). Chloroflexi has many bacterial species that are related to nitrite oxidation (Spieck et al., 2020), while Alphaproteobacteria also contains rhizobacteria that have been widely proven to have N-fixing abilities (Wang et al., 2019). In general, ZZ1 and ZZ9 can form different rhizosphere bacterial communities because of their differences in root morphology, root exudates, and other metabolites, which lead to differences in the utilization of nutrients such as N and P, and form different network structures.

### ZZ9 Dominates the Structure of the Bacterial Community in the Intercropping Pattern

Through co-occurrence network analysis of the two intercropping patterns, we found that the distribution of csOTUs contained in M1 and M2 representing the intercropping treatment of ZZ9 and soybeans was denser, while that of ZZ1 and soybean intercropping treatment was more dispersed (Figures 4A,B). In other words, ZZ9 was more closely related to the rhizosphere bacterial community of soybean, which may be because ZZ9 interacted more closely with the soybean root system than ZZ1. When the biomass of two intercropping species is different, the priority of resource acquisition will be different (Malézieux et al., 2009). The yield data showed that the biomass of ZZ9 was significantly larger than that of ZZ1 (Supplementary Figure 1), indicating that their degree of interaction with soybean upon intercropping was also different. Plant to plant interactions shape ecosystem structure by changing community composition (Kardol et al., 2010). Regarding interactions of legumes, it known that legumes can fix atmospheric N_2_ through symbiotic relationships with rhizobia (Clúa et al., 2018). In addition, ZZ9 had a higher cumulative relative abundance of bacteria than ZZ1 in the modules representing intercropping, which also indicated that ZZ9 interacted more effectively with soybean and could accumulate more csOTUs (Figure 4B); this is also reflected in Figure 4C, which displays a comparison of the number of OTUs (according to taxonomy) in each module, and in Supplementary Figure 1, which shows the result of the node degree analysis. In the keystone analysis, the key species of ZZ1 mostly belonged to the rhizosphere of soybean, whereas the key species of ZZ9 all appeared in its rhizosphere (Supplementary Table 1 and Supplementary Table 2), of which Acidobacteria accounted for the majority. There is clear evidence for the association of some Acidobacteria with plants (Da Rocha et al., 2010, 2013). Acidobacteria strains produce exopolysaccharide (EPS) for the adhesion of bacteria to the root surfaces (Kielak et al., 2016). This indicates that ZZ9 occupies a dominant position in intercropping with soybean and is more closely related to the rhizosphere bacteria. Mastering the advantage of rhizosphere bacteria enrichment is conducive to its own growth and development.

### ZZ9 Improves the Utilization of N, P, and Other Nutrients by Improving Rhizosphere Microbes

The Mantel test was used to check all modules in the network analysis, and it was found that compared with ZZ1, the modules of ZZ9 were more closely related to nutrients, and the relationship among nutrients was also closer (Supplementary Figure 4). In addition, the analysis of soil environmental factors also indicated that ZZ1 and ZZ9 had significant differences in SOC, TN, and AP (Table 1). These microbial communities provide available N to plants through biological N fixation and mineralization of organic forms, and limit N loss by fixing it in soil organic matter (Schmidt et al., 2019). There is little information on plant-microbial interactions affecting P use, but Castrillo et al. (2017) demonstrated that *Arabidopsis* mutants could enrich bacterial communities in phosphate rich soil, compete with plants for phosphate, and induce phosphate fixation. This suggests that plant roots have the potential to assemble specific microbiota, thereby facilitating the P cycle. In addition, the interaction is not only affected by the rhizosphere bacterial community, but also by the enzyme activity of rhizosphere soil (Li et al., 2018b). Soil enzymes are important bioactive proteins in soil, which mainly come from microbes (Zheng et al., 2018). They are directly involved in soil nutrient cycling and are closely related to soil fertility and soil environmental quality (Yang et al., 2018b). Higher soil enzyme activity can improve the effective N and P supply capacity of plant soil (Li et al., 2018b). Our study found that the diversity of ZZ1 rhizosphere bacteria was negatively correlated with S-U (Figure 1B) and was affected by AP, SOC, and S-CAT, while that of ZZ9 was greatly influenced by TN and S-SC (Figure 2B). Urease participates in soil N cycle, reflecting soil quality and fertility (Adetunji et al., 2017); the activity of urease increases with the increase of SOC in soil (Abujabhah et al., 2016), and it has been proven to be correlated with P and S-CAT in soil (Kravkaz Kuscu et al., 2018). Nevertheless, the enhanced P uptake by plants can also be achieved via hormonal stimulation of root growth, i.e., branching or root hair development mediated by indole-3-acetic acid (IAA) among other hormones (Richardson and Simpson, 2011).

Functional annotation was performed on the representative sequences of csOTUs in the network analysis using the PICRUSt2 software, and it was also found that ZZ9 contained more genes related to N and P cycle pathways (Figure 5), like the genes involved in P-uptake and transport, inorganic P-solubilization, and organic P-mineralization; these genes greatly increase the efficiency of plant absorption and utilization of nutrients (Manzoor et al., 2017). In other words, whether in the ZZ9 intercropping treatment, N or P cycle pathways have more response genes or environmental factors and related functional genes have higher contribution (Supplementary Figure 5), the results showed that ZZ9 was able to influence the environmental factors in its rhizosphere more widely, so as to create a more suitable environment for sugarcane and soybean growth (Bossolani et al., 2020); this was consistently displayed by the Pearson analysis (Figures 1B,C) and the Mantel test (Figure 2D and Supplementary Figure 4) in our study. In other words, ZZ9 could better enrich microbial flora in the intercropping process, and use it to promote its own growth and development (Duchene et al., 2017). In addition, the changes in bacterial structure in the rhizosphere may affect the activities of various enzymes in the soil, thus indirectly affecting the nutrient supply in the soil (Li et al., 2018a). Some strains may have no direct effect on the growth of the plant, but on the whole, these bacteria may have some functional roles (such as in N and P cycle pathways) (Marschner et al., 2003), which have advantages in terms of bacterial nutrient absorption and enrichment; these functional microbes are known to form close links and in turn promote plant development via root soil nutrient absorption and enrichment (Vejan et al., 2016; Ilangumaran and Smith, 2017; Etesami and Maheshwari, 2018). In addition, ZZ9 and ZZ1 also showed significant difference in the bacterial contribution of related functional genes in the N and P cycle pathways (Supplementary Figure 6 and Supplementary Figure 7); this was also supported by the results of the bacterial environment factor analysis between the two intercropping patterns (Figure 3B).

## Conclusions

We investigated the difference in rhizosphere bacterial diversity between ZZ1 and ZZ9 in intercropping patterns with soybean, analyzed the difference in csOTUs by applying co-occurrence network analysis, and used PICRUSt2 gene annotation to find the difference in nutrient utilization between the microbiotas of the two sugarcane varieties. Based on the results of this study, we believe that ZZ9 is more suitable for intercropping with soybean than ZZ1. Compared with ZZ1, ZZ9 had higher bacterial diversity in its intercropping with soybean. Further, ZZ9 is more dominant than ZZ1 in the intercropping system with soybean, as it can enrich and promote the growth of the corresponding bacterial community, change the structure of the rhizosphere bacterial community, and improve the absorption and utilization of N, P, and other nutrients by the root system. Thus, the interaction between ZZ9 and legumes has the potential to promote efficient agricultural production, which is of the utmost significance to the sugarcane industry in Guangxi, China. In addition, our findings will help with the design of intercropping systems in future: that is, appropriate varieties should be selected, so as to form an environment conducive to crop growth and to enrich the bacterial community structure that promotes crop growth. This will maximize yield as far as possible.

## Data Availability Statement

The datasets presented in this study can be found in online repositories. The names of the repository/repositories and accession number(s) can be found at: https://www.ncbi.nlm.nih.gov/, PRJNA657992.

## Author Contributions

YL: writing—original draft preparation and investigation. HY: investigation and formal analysis. QL: visualization and data curation. XZ: investigation and formal analysis. SX: software and validation. ZW: writing—review & editing and software. RW: methodology and writing—review & editing. MZ: conceptualization. BC: writing—review & editing.

## Conflict of Interest

The authors declare that the research was conducted in the absence of any commercial or financial relationships that could be construed as a potential conflict of interest.

## Supplementary Material

Supplementary Figure 1 Comparison of field characters between zz1—and zz9.

Supplementary Figure 2 **(A)** Schematic diagram of experiment design. The box represents a planting cell, the gray line represents rows, the star represents sugarcane, and the triangle represents soybean. **(B)** Schematic diagram of the collection site of the rhizosphere soil samples. **(C)** Schematic diagram of the block division and sample selection.

Supplementary Figure 3 Degree of co-occurrence and abundance of csOTUs. Circles refer to bacteria. OTUs were colored by their association to the different cropping systems. Side panels recapitulate the distributions of co-occurrence degrees and abundance for the csOTUs compared to the density of all.

Supplementary Figure 4 Mantel test between each module and soil physicochemical properties in two intercropping models.

Supplementary Figure 5 Genetic quality of representation in N cycle and P cycle. It was used to show the representativeness of each variable in each principal component. A high Cos2 value indicates that the variable has good representation in the principal component. The vertical axis represents the quality of representation, the minimum value is 0 and the maximum value is 1, the larger the value, the higher the quality.

Supplementary Figure 6 The contribution of different Bacteria to N-cycle related functional genes. The colors in the graph represent different phyla, and the horizontal axis represents the contribution of the phylum to the functional genes. The colors in the graph represent different phyla, and the horizontal axis represents the contribution of the phylum to the functional genes.

Supplementary Figure 7 The contribution of different Bacteria to P-cycle related functional genes. The colors in the graph represent different phyla, and the horizontal axis represents the contribution of the phylum to the functional genes. The colors in the graph represent different phyla, and the horizontal axis represents the contribution of the phylum to the functional genes.

Supplementary Figure 8 **(A)** PCA analysis of N-cycle related functional genes and each line. The dot indicates line, and the arrow indicates the function gene. The color of the arrow indicates the function of the gene. The color of the arrow indicates the contribution of the gene. **(B)** PCA analysis of P-cycle related functional genes and each line. The dot indicates line, and the arrow indicates the function gene. The color of the arrow indicates the function of the gene. The color of the arrow indicates the contribution of the gene.

Supplementary Figure 9 Root correlation of ZZ1 sugarcane and ZZ9 sugarcane in the same growing period.

Supplementary Table 1 Contribution of various bacterial phylum.

Supplementary Table 2 ZZ1 keystone.

Supplementary Table 3 ZZ9 keystone.
