# Supplementary figures and images for: Effect of Two Different Sugarcane Cultivars on Rhizosphere Bacterial Communities of Sugarcane and Soybean Upon Intercropping
Source: Front Microbiol. 2021 Jan 14;11:596472. doi: 10.3389/fmicb.2020.596472 (PMC7841398; doi:10.3389/fmicb.2020.596472)

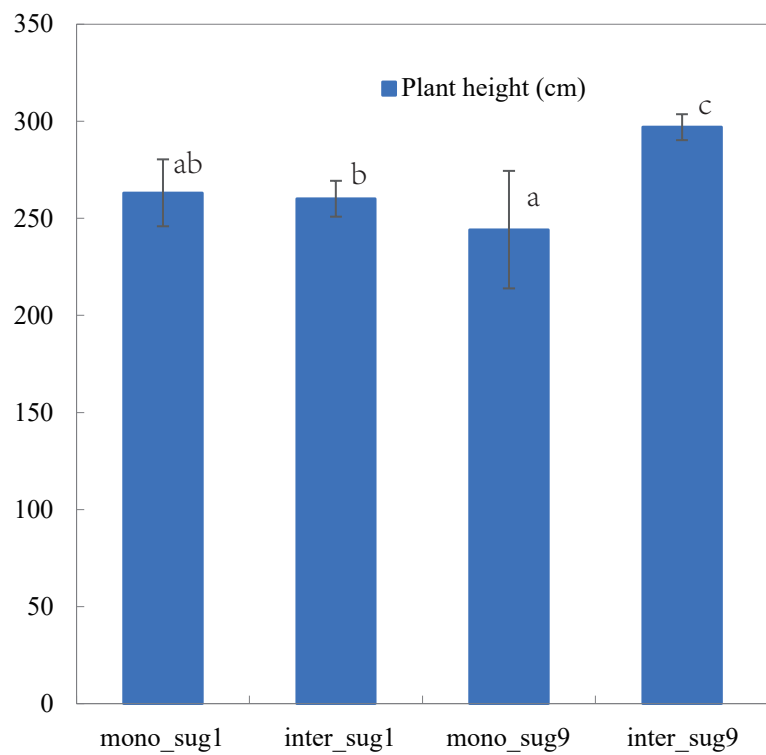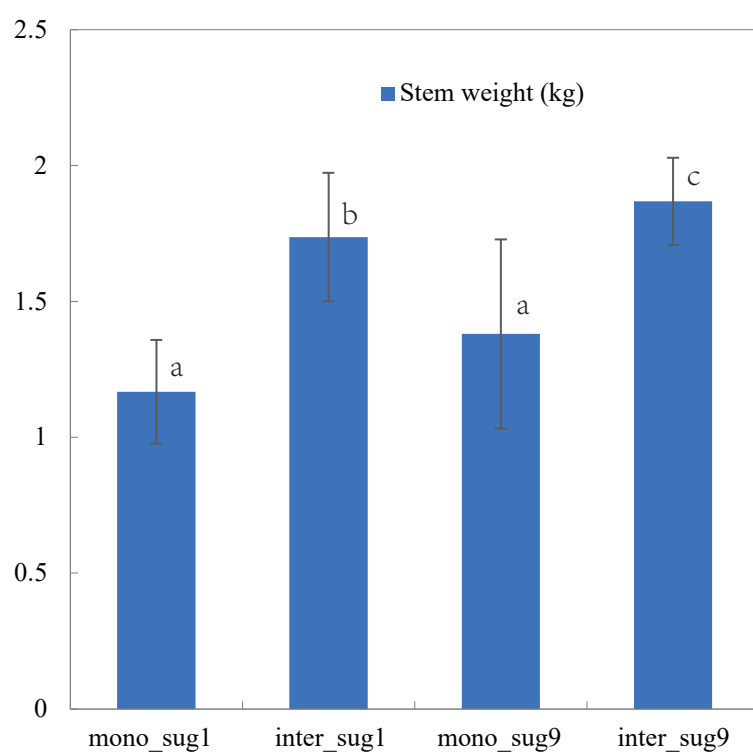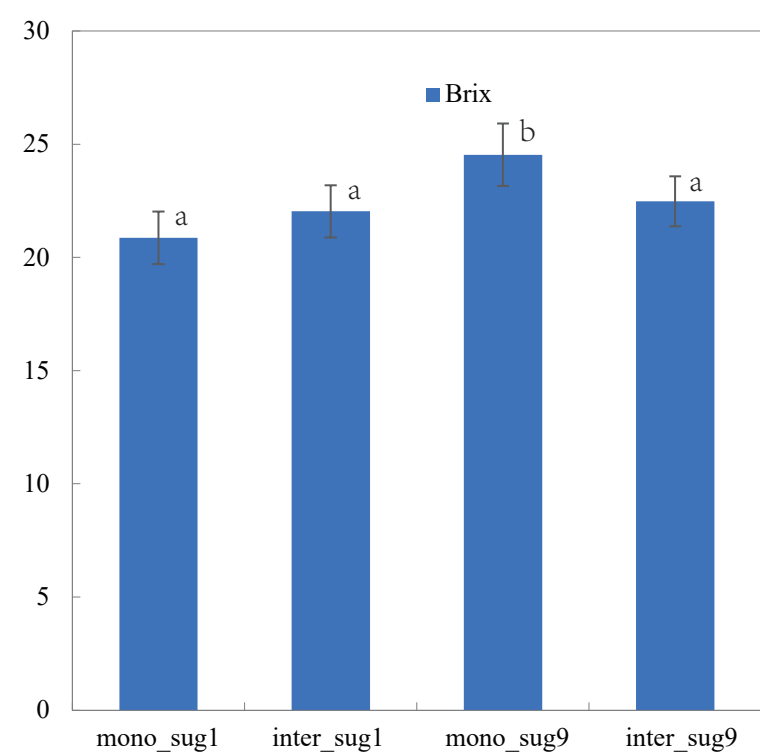

Supplement: Supplementary Figure 1 — Comparison of field characters between zz1—and zz9. [file Data_Sheet_1.PDF]

A)

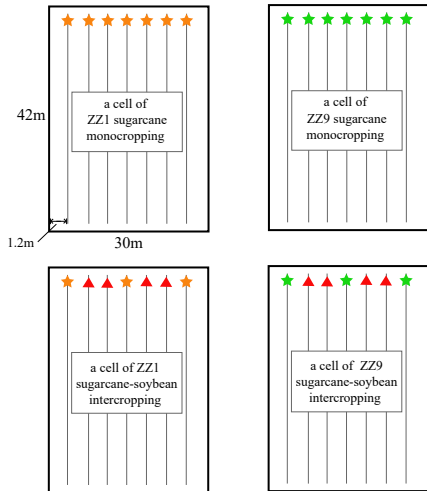

B)

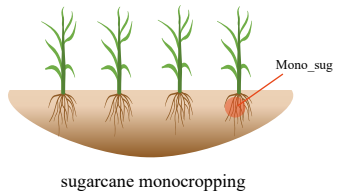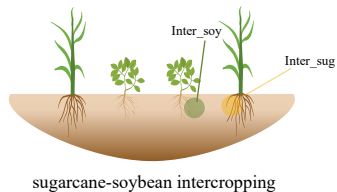

C)

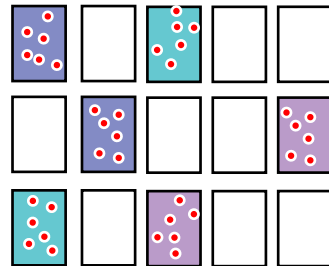

Legends

- ★ — ZZ1 sugarcane line
- ★ — ZZ9 sugarcane line
- ▲ — soybean line
- — sampling point

- block1
- block2
- block3

Supplement: Supplementary Figure 2 — (A) Schematic diagram of experiment design. The box represents a planting cell, the gray line represents rows, the star represents sugarcane, and the triangle represents soybean. (B) Schematic diagram of the collection site of the rhizosphere soil samples. (C) Schematic diagram of the block division and sample selection. [file Data_Sheet_2.PDF]

**ZZ1**

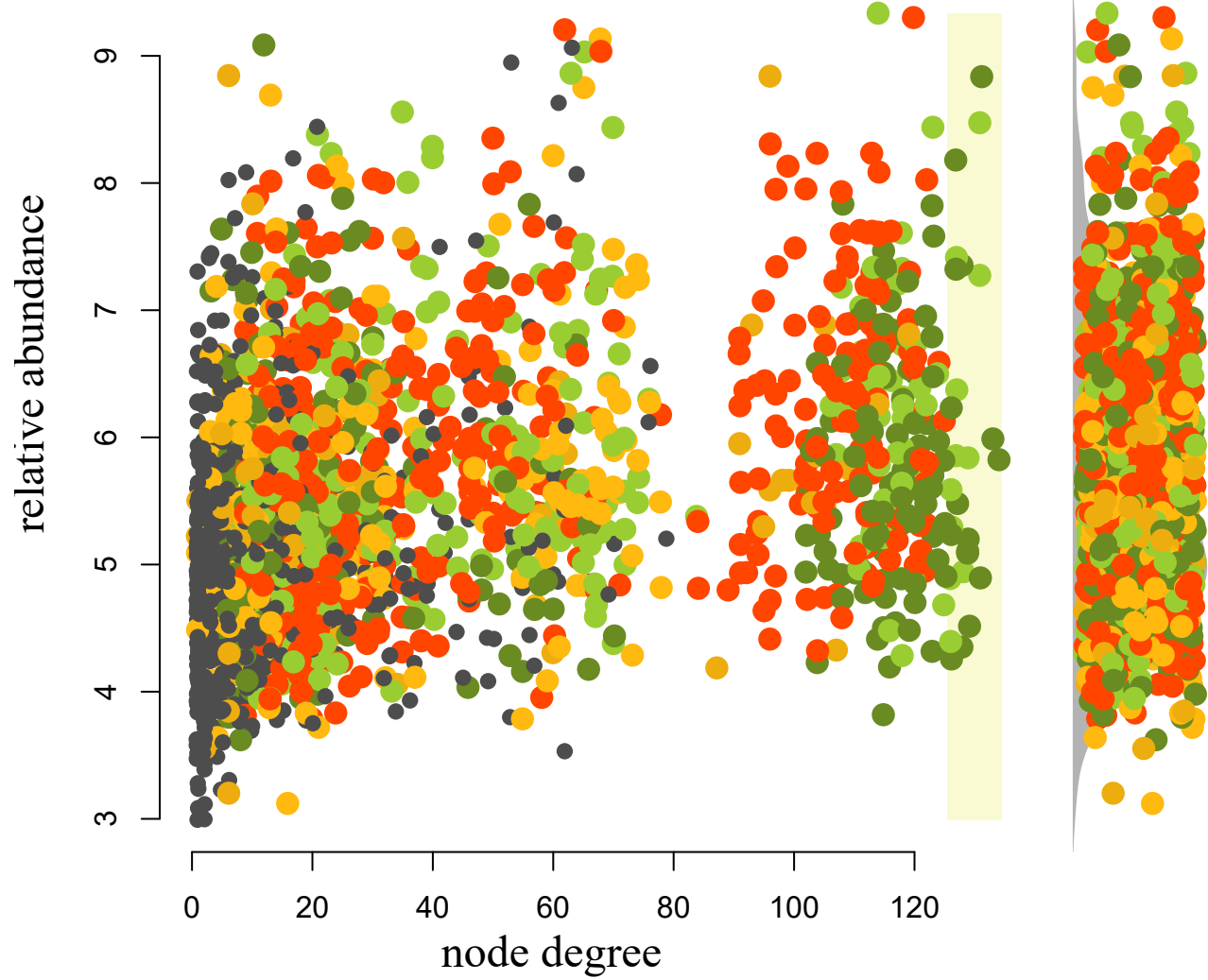

**ZZ9**

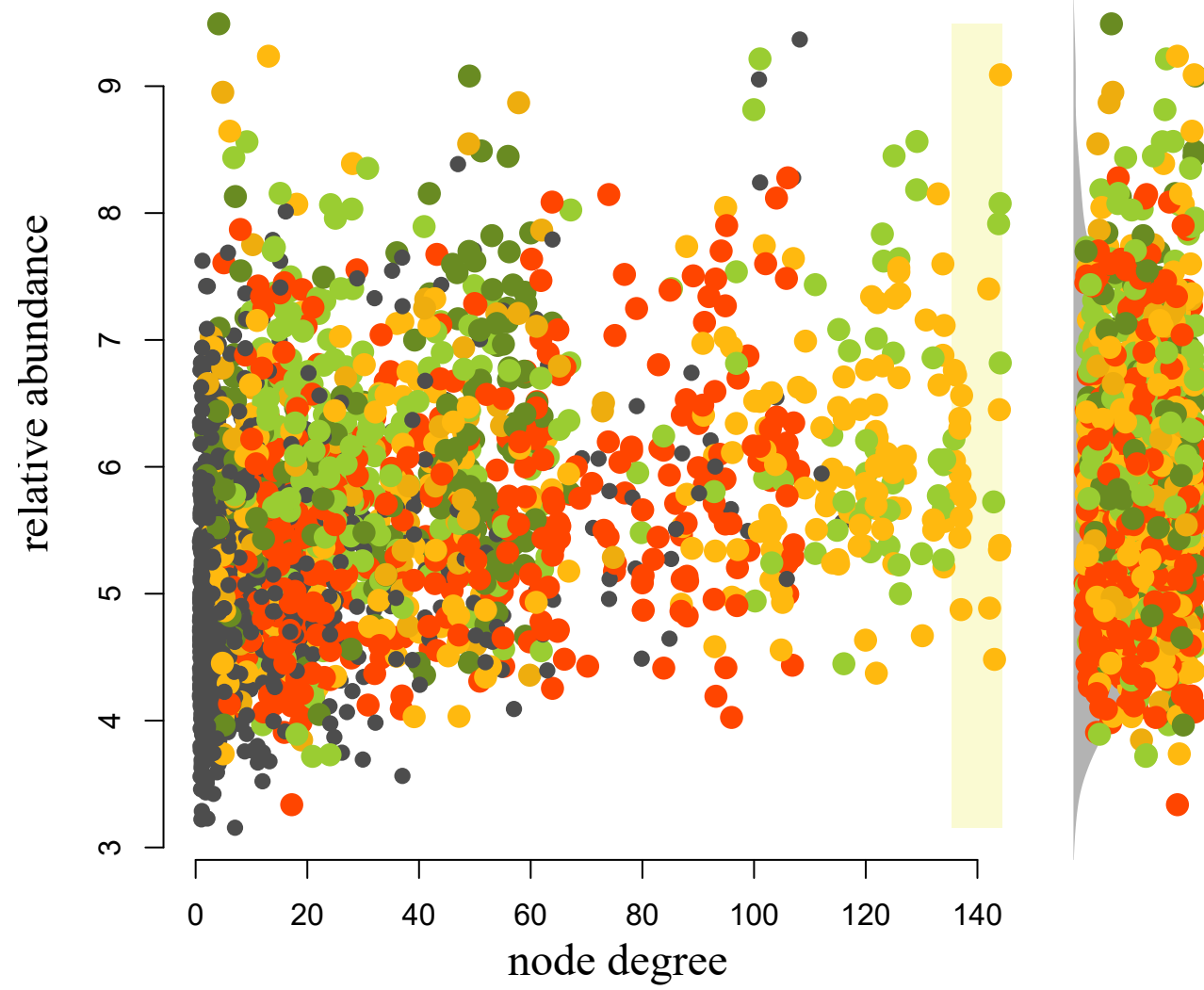

Supplement: Supplementary Figure 3 — Degree of co-occurrence and abundance of csOTUs. Circles refer to bacteria. OTUs were colored by their association to the different cropping systems. Side panels recapitulate the distributions of co-occurrence degrees and abundance for the csOTUs compared to the density of all. [file Data_Sheet_3.PDF]

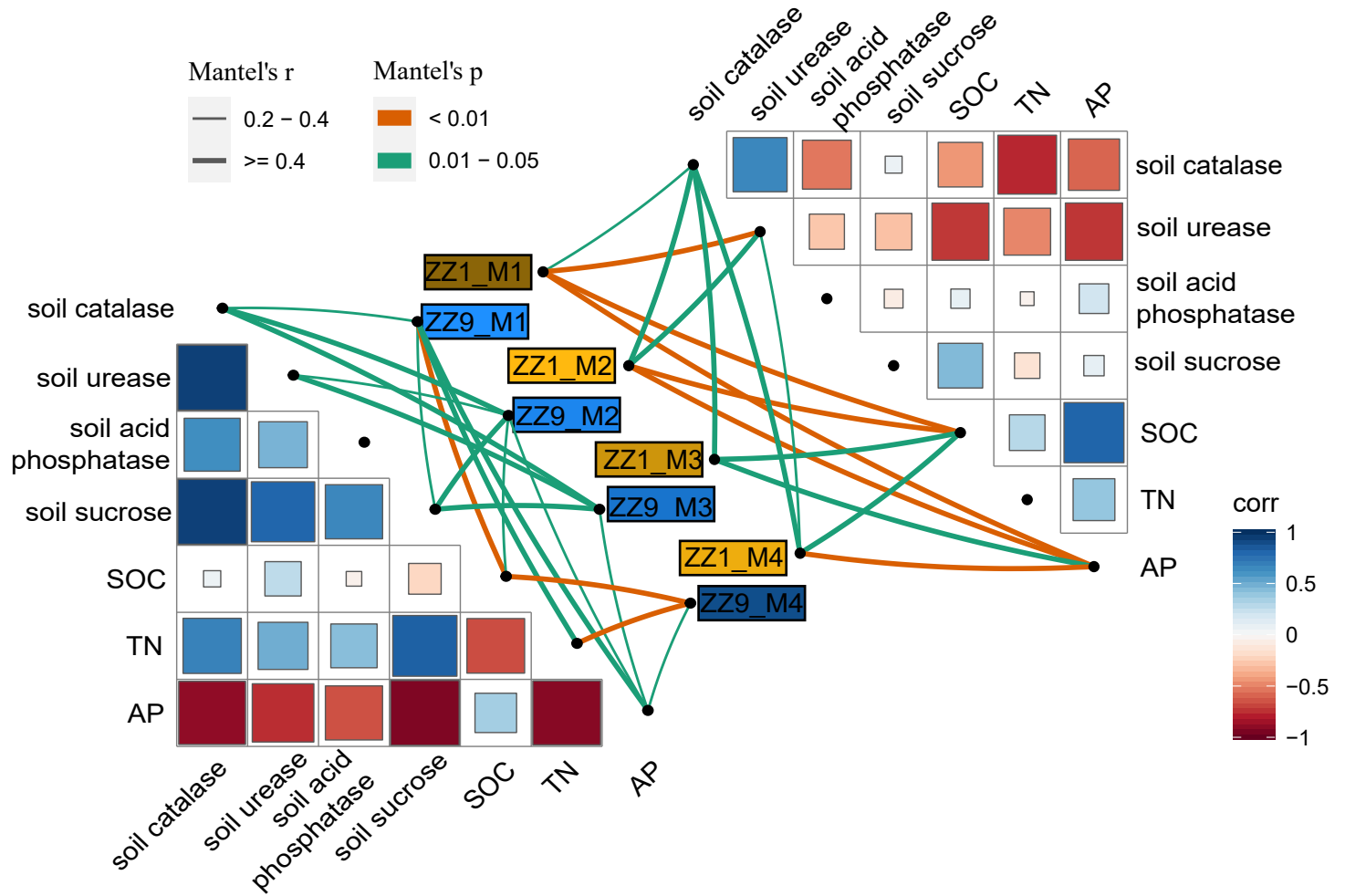

Supplement: Supplementary Figure 4 — Mantel test between each module and soil physicochemical properties in two intercropping models. [file Data_Sheet_4.PDF]

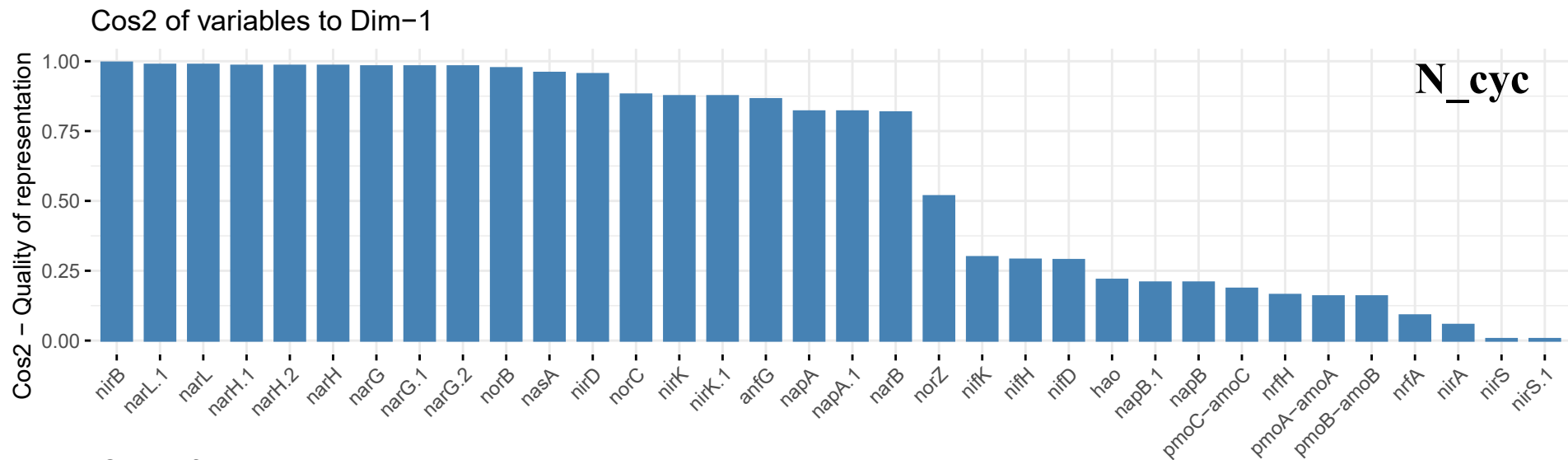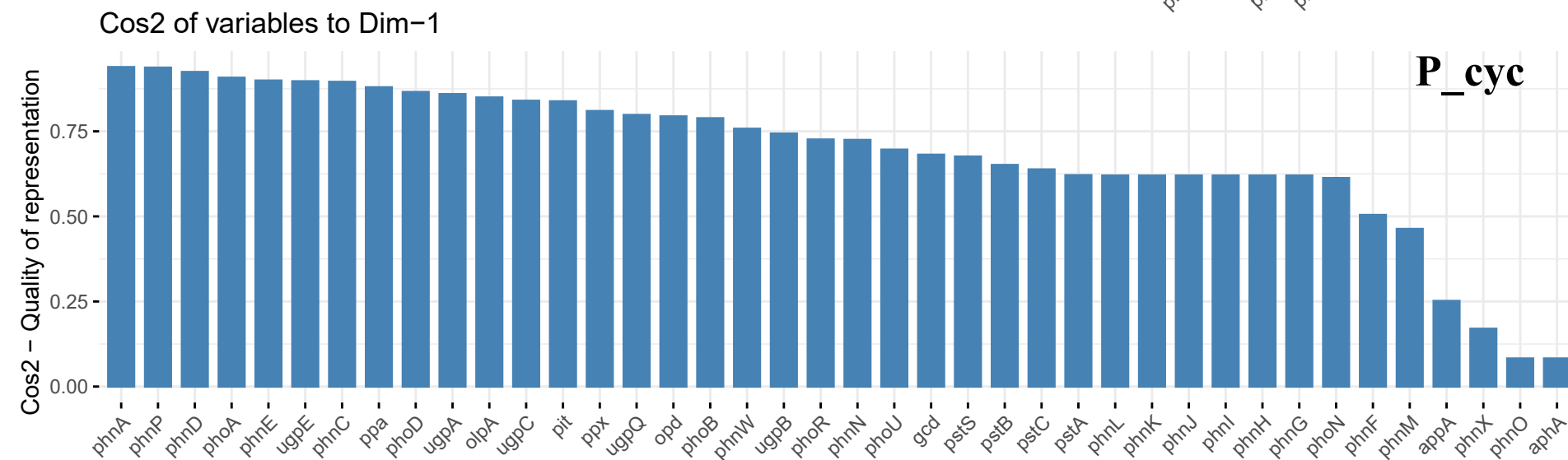

Supplement: Supplementary Figure 5 — Genetic quality of representation in N cycle and P cycle. It was used to show the representativeness of each variable in each principal component. A high Cos2 value indicates that the variable has good representation in the principal component. The vertical axis represents the quality of representation, the minimum value is 0 and the maximum value is 1, the larger the value, the higher the quality. [file Data_Sheet_5.PDF]

N-CYC-PHY

gene

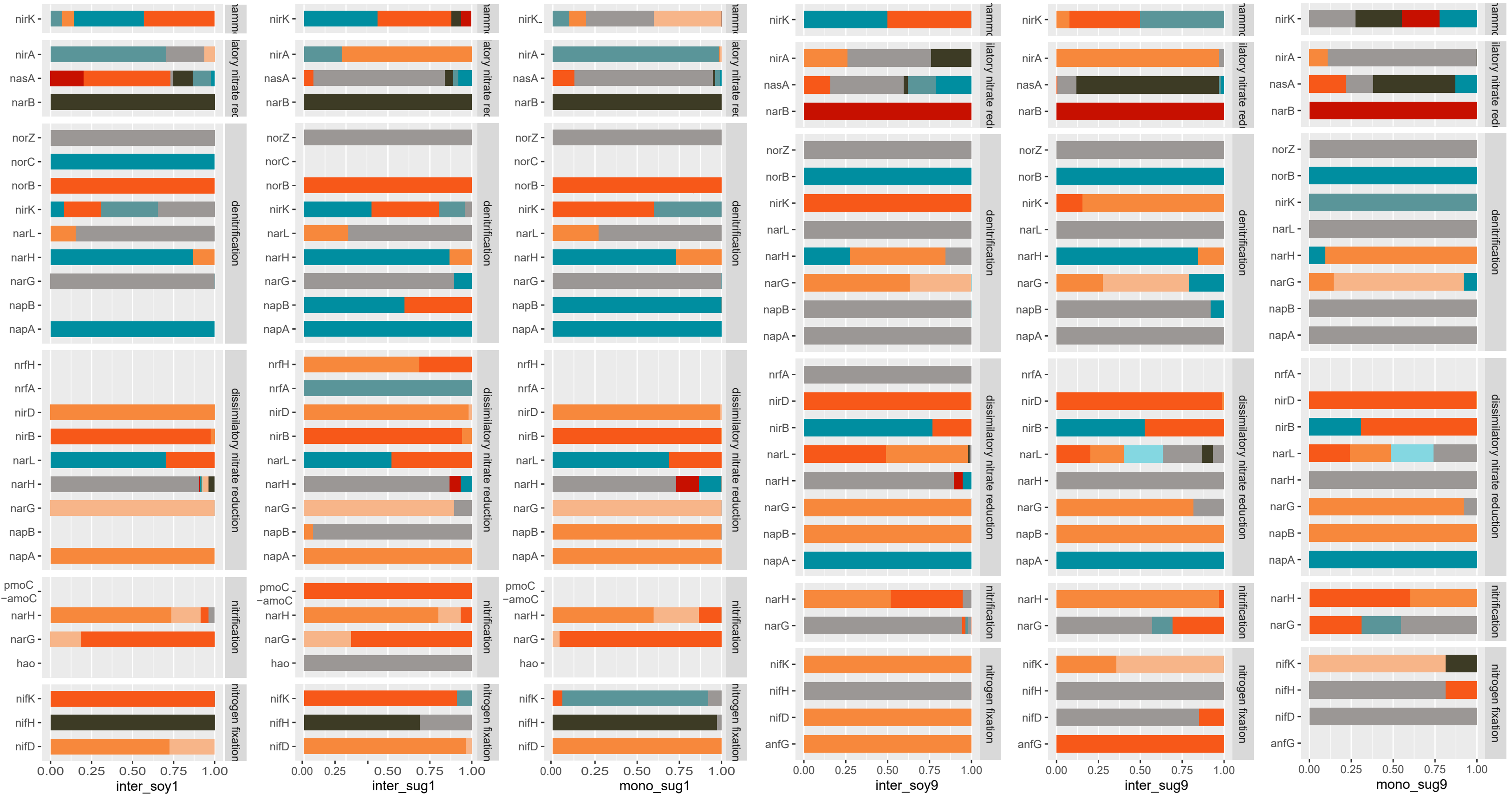

Supplement: Supplementary Figure 6 — The contribution of different Bacteria to N-cycle related functional genes. The colors in the graph represent different phyla, and the horizontal axis represents the contribution of the phylum to the functional genes. The colors in the graph represent different phyla, and the horizontal axis represents the contribution of the phylum to the functional genes. [file Data_Sheet_6.PDF]

P-CYC-PHY

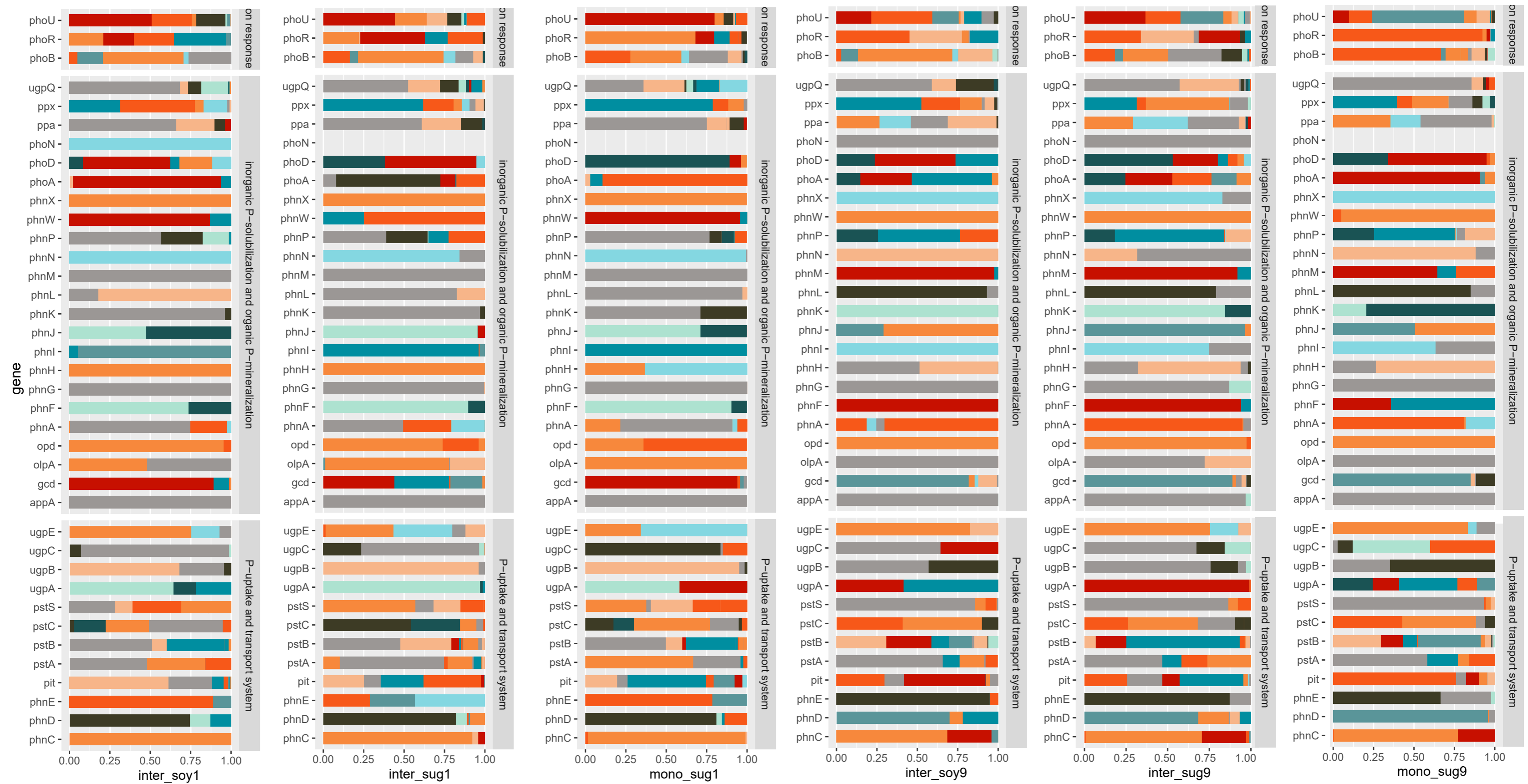

Supplement: Supplementary Figure 7 — The contribution of different Bacteria to P-cycle related functional genes. The colors in the graph represent different phyla, and the horizontal axis represents the contribution of the phylum to the functional genes. The colors in the graph represent different phyla, and the horizontal axis represents the contribution of the phylum to the functional genes. [file Data_Sheet_7.PDF]

**A) PCA – Biplot – N cycling**

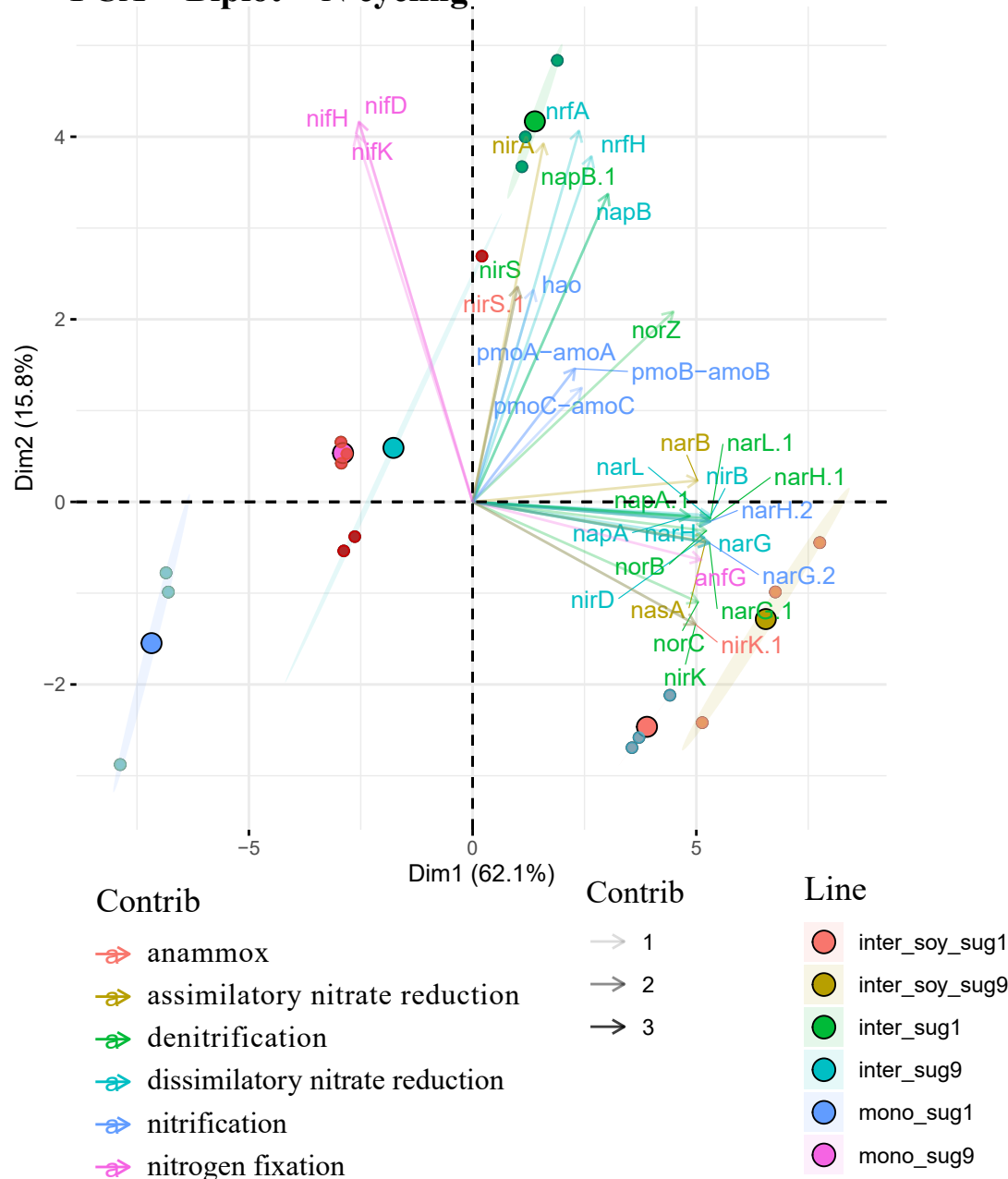

**B) PCA – Biplot – P cycling**

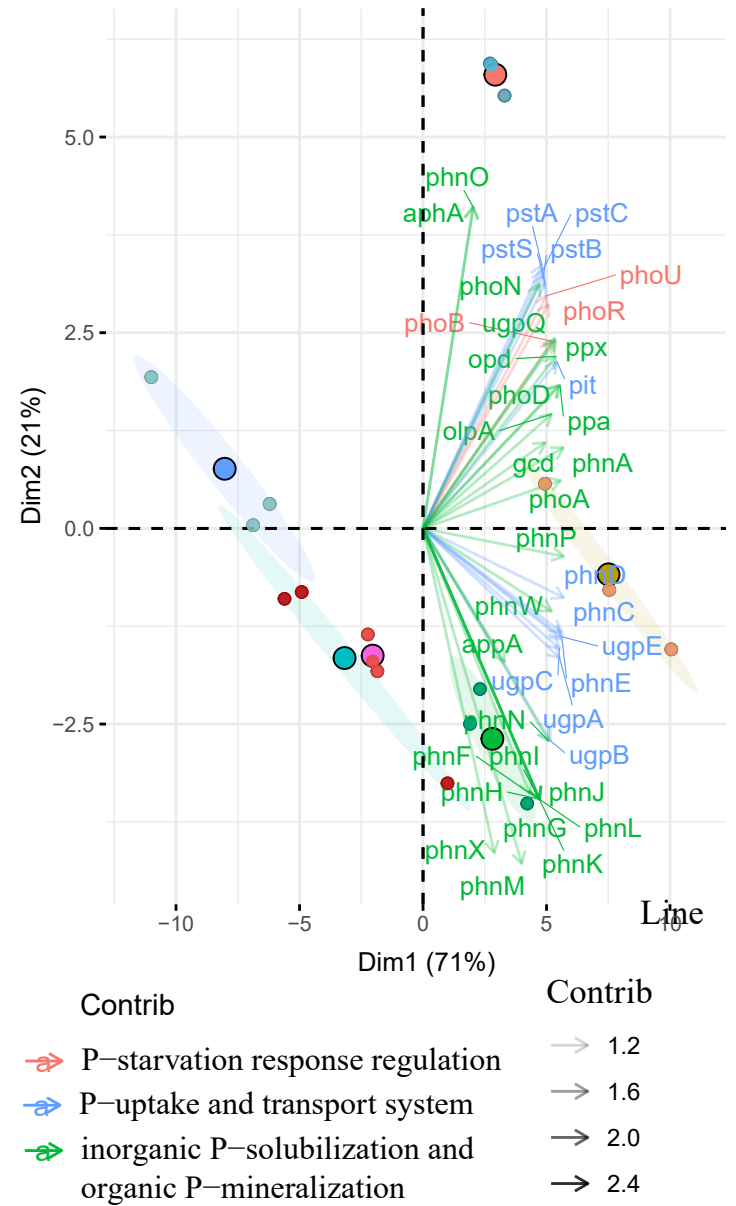

Supplement: Supplementary Figure 8 — (A) PCA analysis of N-cycle related functional genes and each line. The dot indicates line, and the arrow indicates the function gene. The color of the arrow indicates the function of the gene. The color of the arrow indicates the contribution of the gene. (B) PCA analysis of P-cycle related functional genes and each line. The dot indicates line, and the arrow indicates the function gene. The color of the arrow indicates the function of the gene. The color of the arrow indicates the contribution of the gene. [file Data_Sheet_8.PDF]

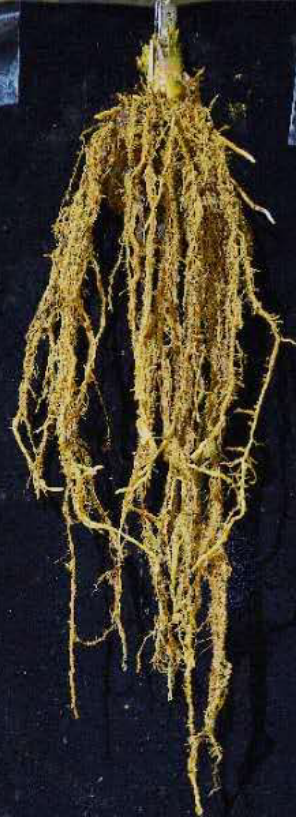

ZZ1

中藥  
1号  
(10)

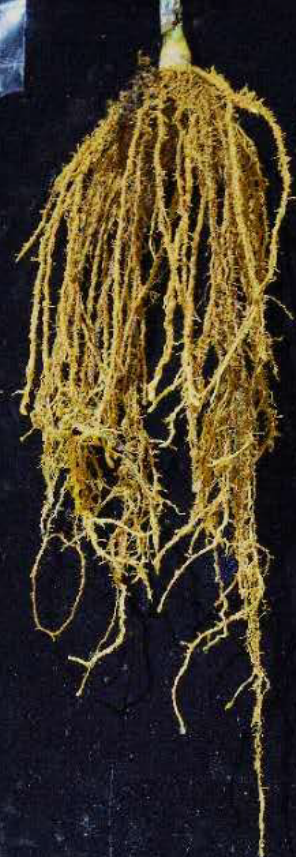

ZZ9

中藥  
6号  
(13)

Supplement: Supplementary Figure 9 — Root correlation of ZZ1 sugarcane and ZZ9 sugarcane in the same growing period. [file Data_Sheet_9.PDF]
