# Supplementary material for: Effect of Two Different Sugarcane Cultivars on Rhizosphere Bacterial Communities of Sugarcane and Soybean Upon Intercropping
Source: Front Microbiol. 2021 Jan 14;11:596472. doi: 10.3389/fmicb.2020.596472 (PMC7841398; doi:10.3389/fmicb.2020.596472)
Supplement: Supplementary Table 1 — Contribution of various bacterial phylum. [file Table_1.DOCX]

| **Table S1 Contribution of various bacterial phylum** | | | | | | | | | | | | |
| --- | --- | --- | --- | --- | --- | --- | --- | --- | --- | --- | --- | --- |
|  | **sample_name** | **Acidobacteria** | **Chloroflexi** | **Actinobacteria** | **Bacteroidetes** | **Proteobacteria** | | | **Verrucomicrobia** | **Planctomycetes** | **WD272** | **Others** |
|  |  |  |  |  |  | **Alphaproteobacteria** | **Gammaproteobacteria** | **Betaproteobacteria** |  |  |  |  |
| 1 | DU1J1 | 21.78322 | 24.41316 | 17.04947 | 4.0575 | 12.72928 | 3.803237 | 4.560667 | 0.714543 | 2.665176 | 2.951606 | 5.272147 |
| 2 | DU1J2 | 21.2739 | 23.48339 | 18.66533 | 2.973009 | 13.01685 | 4.553576 | 4.175752 | 0.657258 | 2.229167 | 3.590123 | 5.381642 |
| 3 | DU1J3 | 21.55826 | 22.49074 | 17.48812 | 4.842838 | 13.72402 | 4.163951 | 4.810245 | 0.625626 | 1.951603 | 2.89203 | 5.452565 |
| 4 | DU9J1 | 21.77374 | 19.54391 | 16.9312 | 4.974109 | 16.28856 | 2.84042 | 5.350245 | 1.378886 | 2.460067 | 3.208123 | 5.25073 |
| 5 | DU9J2 | 22.66717 | 18.28346 | 17.31481 | 5.10306 | 16.83349 | 3.182279 | 5.458983 | 1.148864 | 1.846443 | 2.591327 | 5.570115 |
| 6 | DU9J3 | 26.05219 | 16.98296 | 17.00422 | 4.835504 | 16.33219 | 2.828645 | 4.861387 | 1.063977 | 1.854334 | 2.929404 | 5.255179 |
| 7 | ZZ1D1 | 23.24045 | 13.53529 | 12.91884 | 7.111136 | 16.8155 | 5.053388 | 5.084651 | 4.586847 | 3.411678 | 1.664155 | 6.578061 |
| 8 | ZZ1D2 | 25.90584 | 12.82439 | 12.1604 | 6.728263 | 16.17622 | 5.511788 | 4.790593 | 4.030296 | 3.997712 | 1.820367 | 6.054133 |
| 9 | ZZ1D3 | 24.66678 | 12.25965 | 14.1791 | 6.135371 | 17.57779 | 6.000089 | 5.474485 | 3.083764 | 3.017232 | 1.561287 | 6.044443 |
| 10 | ZZ1J1 | 27.41717 | 19.72559 | 11.64871 | 2.883242 | 15.02641 | 3.878019 | 3.344736 | 3.358654 | 2.859068 | 3.152813 | 6.705588 |
| 11 | ZZ1J2 | 29.11919 | 19.84265 | 10.69419 | 3.239287 | 13.64219 | 3.460314 | 2.970688 | 4.716215 | 3.076079 | 3.128774 | 6.11044 |
| 12 | ZZ1J3 | 28.52847 | 18.72226 | 12.96052 | 2.648324 | 15.35329 | 3.885174 | 3.55211 | 2.736062 | 2.398739 | 2.726692 | 6.488351 |
| 13 | ZZ9D1 | 22.25754 | 10.62866 | 15.62122 | 6.330831 | 21.00068 | 3.968303 | 7.037962 | 2.894427 | 2.273362 | 1.361536 | 6.625469 |
| 14 | ZZ9D2 | 21.52147 | 10.70553 | 15.42927 | 6.296486 | 21.27854 | 3.860078 | 6.46922 | 3.796979 | 2.505817 | 1.624798 | 6.511811 |
| 15 | ZZ9D3 | 23.85021 | 9.766613 | 15.31634 | 6.234679 | 21.13328 | 4.258656 | 6.680014 | 2.974675 | 1.990999 | 1.317874 | 6.476657 |
| 16 | ZZ9J1 | 28.69512 | 20.30718 | 10.80315 | 3.43786 | 15.6502 | 2.319212 | 5.304621 | 3.846154 | 1.945475 | 1.529502 | 6.161526 |
| 17 | ZZ9J2 | 30.15631 | 18.98826 | 12.04314 | 2.408436 | 15.5205 | 2.096587 | 4.889797 | 2.98032 | 2.299049 | 2.44298 | 6.174616 |
| 18 | ZZ9J3 | 27.60931 | 18.69938 | 12.57544 | 2.915556 | 17.37853 | 2.671189 | 5.582532 | 2.824972 | 1.925447 | 1.787464 | 6.030188 |
|  | average | 24.89313 | 17.28906 | 14.48908 | 4.61975 | 16.41542 | 3.796384 | 5.022149 | 2.634362 | 2.483747 | 2.348936 | 6.007981 |
