# Supplementary material for: Effect of Two Different Sugarcane Cultivars on Rhizosphere Bacterial Communities of Sugarcane and Soybean Upon Intercropping
Source: Front Microbiol. 2021 Jan 14;11:596472. doi: 10.3389/fmicb.2020.596472 (PMC7841398; doi:10.3389/fmicb.2020.596472)
Supplement: Supplementary Table 2 — ZZ1 keystone. [file Table_2.DOCX]

| **Table S2 ZZ1 keystone** | | | | | | | | | | | | |
| --- | --- | --- | --- | --- | --- | --- | --- | --- | --- | --- | --- | --- |
| otuid | s.inter  soy1 | s.inter  sug1 | s.mono  sug1 | stat | p.value | Kingdom | Phylum | Class | Order | Family | Genus | Species |
| OTU_980 | 1 | 0 | 0 | 0.981295 | 0.037 | Bacteria | Gammaproteobacteria | Gammaproteobacteria | Xanthomonadales | Xanthomonadaceae | Unassigned | Unassigned |
| OTU_627 | 1 | 0 | 0 | 0.869577 | 0.037 | Bacteria | Actinobacteria | Thermoleophilia | Gaiellales | Unassigned | Unassigned | Unassigned |
| OTU_109 | 1 | 1 | 0 | 0.908608 | 0.026 | Bacteria | Acidobacteria | Acidobacteria | Acidobacteriales | Acidobacteriaceae_[Subgroup_1] | Unassigned | Unassigned |
| OTU_14 | 1 | 0 | 0 | 0.911855 | 0.0338 | Bacteria | Acidobacteria | Acidobacteria | Subgroup_3 | Unknown_Family | Bryobacter | Unassigned |
| OTU_2875 | 1 | 0 | 0 | 0.852977 | 0.0299 | Bacteria | Actinobacteria | Actinobacteria | Micrococcales | Intrasporangiaceae | Unassigned | Unassigned |
| OTU_9 | 1 | 1 | 0 | 0.855006 | 0.0259 | Bacteria | Acidobacteria | Acidobacteria | Subgroup_3 | Unknown_Family | Bryobacter | Unassigned |
| OTU_1019 | 1 | 1 | 0 | 0.833887 | 0.0052 | Bacteria | Planctomycetes | Planctomycetacia | Planctomycetales | Planctomycetaceae | Unassigned | Unassigned |
| OTU_1182 | 1 | 0 | 0 | 0.788654 | 0.037 | Bacteria | Alphaproteobacteria | Alphaproteobacteria | Rhodospirillales | Acetobacteraceae | Acidicaldus | Unassigned |
| OTU_1360 | 1 | 0 | 0 | 0.834381 | 0.037 | Bacteria | Chloroflexi | Ktedonobacteria | Ktedonobacterales | FCPS473 | Unassigned | Unassigned |
| OTU_2582 | 1 | 0 | 0 | 0.910992 | 0.037 | Bacteria | Alphaproteobacteria | Alphaproteobacteria | Rhodospirillales | Acetobacteraceae | Acidicaldus | Unassigned |
| OTU_4234 | 1 | 0 | 0 | 0.869338 | 0.037 | Bacteria | Chloroflexi | JG37-AG-4 | Unassigned | Unassigned | Unassigned | Unassigned |
| OTU_1334 | 1 | 1 | 0 | 0.872805 | 0.0356 | Bacteria | Actinobacteria | Acidimicrobiia | Acidimicrobiales | Unassigned | Unassigned | Unassigned |
| OTU_1407 | 1 | 0 | 0 | 0.797073 | 0.037 | Bacteria | Chloroflexi | TK10 | Unassigned | Unassigned | Unassigned | Unassigned |
| OTU_427 | 1 | 0 | 0 | 0.951591 | 0.0155 | Bacteria | Bacteroidetes | Sphingobacteriia | Sphingobacteriales | Sphingobacteriaceae | Mucilaginibacter | Unassigned |
| OTU_1201 | 1 | 1 | 0 | 0.909193 | 0.0249 | Bacteria | Actinobacteria | Thermoleophilia | Gaiellales | Unassigned | Unassigned | Unassigned |
| OTU_1311 | 1 | 1 | 0 | 0.928554 | 0.0128 | Bacteria | Betaproteobacteria | Betaproteobacteria | Burkholderiales | Burkholderiaceae | Burkholderia | Unassigned |
| OTU_1780 | 1 | 0 | 0 | 0.950582 | 0.0228 | Bacteria | Acidobacteria | Acidobacteria | Subgroup_13 | Unassigned | Unassigned | Unassigned |
| OTU_36 | 1 | 0 | 0 | 0.89051 | 0.037 | Bacteria | Chloroflexi | TK10 | Unassigned | Unassigned | Unassigned | Unassigned |
| OTU_53 | 1 | 1 | 0 | 0.950939 | 0.0313 | Bacteria | Acidobacteria | Acidobacteria | Acidobacteriales | Acidobacteriaceae_[Subgroup_1] | Unassigned | Unassigned |
| OTU_68 | 1 | 0 | 0 | 0.894852 | 0.037 | Bacteria | Alphaproteobacteria | Alphaproteobacteria | Rhodospirillales | Rhodospirillales_Incertae_Sedis | Reyranella | Unassigned |
