# Supplementary material for: Effect of Two Different Sugarcane Cultivars on Rhizosphere Bacterial Communities of Sugarcane and Soybean Upon Intercropping
Source: Front Microbiol. 2021 Jan 14;11:596472. doi: 10.3389/fmicb.2020.596472 (PMC7841398; doi:10.3389/fmicb.2020.596472)
Supplement: Supplementary Table 3 — ZZ9 keystone. [file Table_3.DOCX]

| **Table S3 ZZ9 keystone** | | | | | | | | | | | | |
| --- | --- | --- | --- | --- | --- | --- | --- | --- | --- | --- | --- | --- |
| otuid | s.inter  soy9 | s.inter  sug9 | s.mono  sug9 | stat | p.value | Kingdom | Phylum | Class | Order | Family | Genus | Species |
| OTU_164 | 1 | 1 | 0 | 0.877232 | 0.029 | Bacteria | Acidobacteria | Acidobacteria | Acidobacteriales | Acidobacteriaceae_[Subgroup_1] | Unassigned | Unassigned |
| OTU_2073 | 0 | 1 | 0 | 0.879631 | 0.0355 | Bacteria | Acidobacteria | Acidobacteria | Acidobacteriales | Acidobacteriaceae_[Subgroup_1] | Unassigned | Unassigned |
| OTU_2421 | 0 | 1 | 0 | 0.887991 | 0.0355 | Bacteria | Acidobacteria | Acidobacteria | Subgroup_13 | Unassigned | Unassigned | Unassigned |
| OTU_573 | 1 | 1 | 0 | 0.893212 | 0.0317 | Bacteria | Acidobacteria | Acidobacteria | Acidobacteriales | Acidobacteriaceae_[Subgroup_1] | Unassigned | Unassigned |
| OTU_684 | 0 | 1 | 0 | 0.937013 | 0.0355 | Bacteria | Acidobacteria | Acidobacteria | Acidobacteriales | Acidobacteriaceae_[Subgroup_1] | Unassigned | Unassigned |
| OTU_92 | 1 | 1 | 0 | 0.891128 | 0.0137 | Bacteria | Acidobacteria | Acidobacteria | Acidobacteriales | Acidobacteriaceae_[Subgroup_1] | Unassigned | Unassigned |
| OTU_10 | 0 | 1 | 0 | 0.929057 | 0.0355 | Bacteria | Acidobacteria | Acidobacteria | Acidobacteriales | Acidobacteriaceae_[Subgroup_1] | Unassigned | Unassigned |
| OTU_3345 | 0 | 1 | 0 | 0.848292 | 0.0355 | Bacteria | Acidobacteria | Acidobacteria | Acidobacteriales | Acidobacteriaceae_[Subgroup_1] | Unassigned | Unassigned |
| OTU_552 | 1 | 1 | 0 | 0.789955 | 0.0312 | Bacteria | Chloroflexi | Ktedonobacteria | Ktedonobacterales | Ktedonobacteraceae | Unassigned | Unassigned |
| OTU_267 | 0 | 1 | 0 | 0.951635 | 0.0355 | Bacteria | Betaproteobacteria | Betaproteobacteria | Neisseriales | Neisseriaceae | Unassigned | Unassigned |
| OTU_3276 | 0 | 1 | 0 | 0.835095 | 0.0355 | Bacteria | Chloroflexi | JG37-AG-4 | Unassigned | Unassigned | Unassigned | Unassigned |
| OTU_895 | 0 | 1 | 0 | 0.977758 | 0.0355 | Bacteria | Acidobacteria | Acidobacteria | Subgroup_2 | Unassigned | Unassigned | Unassigned |
| OTU_1199 | 0 | 1 | 0 | 0.958359 | 0.0355 | Bacteria | Acidobacteria | Acidobacteria | Subgroup_3 | Unknown_Family | Candidatus_Solibacter | Unassigned |
| OTU_1369 | 0 | 1 | 0 | 0.943146 | 0.0355 | Bacteria | Acidobacteria | Acidobacteria | Acidobacteriales | Acidobacteriaceae_[Subgroup_1] | Unassigned | Unassigned |
| OTU_1687 | 0 | 1 | 0 | 0.972039 | 0.0355 | Bacteria | Chloroflexi | Ktedonobacteria | Ktedonobacterales | Ktedonobacteraceae | Unassigned | Unassigned |
| OTU_1711 | 0 | 1 | 0 | 0.954866 | 0.0355 | Bacteria | Acidobacteria | Acidobacteria | Acidobacteriales | Acidobacteriaceae_[Subgroup_1] | Unassigned | Unassigned |
| OTU_1748 | 0 | 1 | 0 | 0.943539 | 0.0086 | Bacteria | Bacteroidetes | Sphingobacteriia | Sphingobacteriales | Chitinophagaceae | Unassigned | Unassigned |
| OTU_3360 | 0 | 1 | 0 | 0.888989 | 0.0355 | Bacteria | Acidobacteria | Acidobacteria | Subgroup_3 | Unknown_Family | Bryobacter | Unassigned |
| OTU_605 | 0 | 1 | 0 | 0.864602 | 0.0355 | Bacteria | Acidobacteria | Acidobacteria | Subgroup_3 | Unknown_Family | Candidatus_Solibacter | Unassigned |
| OTU_964 | 0 | 1 | 0 | 0.952118 | 0.0169 | Bacteria | Alphaproteobacteria | Alphaproteobacteria | Rhizobiales | Rhizobiales_Incertae_Sedis | Bauldia | Unassigned |
